# Retinoic Acid Functions as a Key GABAergic Differentiation Signal in the Basal Ganglia

**DOI:** 10.1371/journal.pbio.1000609

**Published:** 2011-04-12

**Authors:** Christina Chatzi, Thomas Brade, Gregg Duester

**Affiliations:** Sanford-Burnham Medical Research Institute, Development and Aging Program, La Jolla, California, United States of America; Stanford University, United States of America

## Abstract

Although retinoic acid (RA) has been implicated as an extrinsic signal regulating forebrain neurogenesis, the processes regulated by RA signaling remain unclear. Here, analysis of retinaldehyde dehydrogenase mutant mouse embryos lacking RA synthesis demonstrates that RA generated by *Raldh3* in the subventricular zone of the basal ganglia is required for GABAergic differentiation, whereas RA generated by *Raldh2* in the meninges is unnecessary for development of the adjacent cortex. Neurospheres generated from the lateral ganglionic eminence (LGE), where *Raldh3* is highly expressed, produce endogenous RA, which is required for differentiation to GABAergic neurons. In *Raldh3*−/− embryos, LGE progenitors fail to differentiate into either GABAergic striatal projection neurons or GABAergic interneurons migrating to the olfactory bulb and cortex. We describe conditions for RA treatment of human embryonic stem cells that result in efficient differentiation to a heterogeneous population of GABAergic interneurons without the appearance of GABAergic striatal projection neurons, thus providing an in vitro method for generation of GABAergic interneurons for further study. Our observation that endogenous RA is required for generation of LGE-derived GABAergic neurons in the basal ganglia establishes a key role for RA signaling in development of the forebrain.

## Abstract 2

Retinoic acid (RA) is essential for the generation of GABAergic inhibitory neurons in the mouse forebrain, and RA treatment of embryonic stem cells induces the production of GABAergic neurons.

## Author Summary

The vitamin A metabolite retinoic acid is an important signaling molecule needed for development of the central nervous system. Previous studies have shown a role for retinoic acid in regulating genes involved in the generation of motor neurons both in the hindbrain and spinal cord, but the role of retinoic acid in the forebrain has remained elusive. Here, we investigated mice that lack the ability to metabolize vitamin A into retinoic acid in the forebrain. Although no defects were observed in the generation of forebrain cortical neurons, we did observe a serious deficiency in GABAergic neurons, which provide inhibitory input to cortical neurons. Specifically, our results reveal that retinoic acid is required for forebrain neurons to activate an enzyme that converts glutamate to the inhibitory neurotransmitter GABA. We also find that retinoic acid treatment of human embryonic stem cells could stimulate production of GABAergic neurons. Deficiencies in GABAergic neurons have been associated with several neurological disorders, including Huntington's disease, autism, schizophrenia, and epilepsy. Knowledge of how GABAergic neurons are generated may aid efforts to treat these diseases.

## Introduction

The embryonic forebrain, deriving from the most anterior part of the neural tube, comprises a complex set of structures in the developing brain. This complexity arises mainly due to the heterogeneity of the neurons comprising it in terms of morphology, structure, function, and genetic specification. During forebrain development, the dorsal domain (pallium) gives rise to the cortex while the ventral region (subpallium) generates the basal ganglia, i.e. the pallidum and the striatum, which, respectively, originate from the medial and lateral ganglionic eminences (MGE, LGE) [1]. The progenitor zones of the subpallial ganglionic eminences are the origin of chemically diverse populations of gamma-aminobutyric acid (GABA)ergic interneurons and projection neurons. GABAergic interneurons are inhibitory local circuit neurons modulating neuronal activity and synaptic plasticity. GABAergic neurons comprise ∼20% of all neurons within the cortex and hippocampus and ∼95% of the neurons within the striatum [2]–[4]. Whereas GABAergic projection neurons generated in the germinal zones of the LGE migrate radially to the adjacent striatum, GABAergic interneurons arise from both the MGE and LGE and migrate using multiple tangential routes to the olfactory bulb, cortex, and hippocampus [5]–[8]. Disturbed GABAergic neuron function has been associated with several neurological disorders including Huntington's disease, autism, schizophrenia, bipolar depression, and epilepsy [9]–[12]. Thus, a source of GABAergic neurons for cell replacement therapy may be useful for treatment of these neurological diseases.

GABAergic neuronal diversity emerges during embryogenesis and depends on both the timing and the creation of specific anteroposterior and dorsoventral progenitor domains by the coordinated action of several transcription factors expressed by distinct progenitor populations [13],[14]. In contrast, little is known about the extrinsic signaling pathways coordinating GABAergic specification in the basal ganglia. Retinoic acid (RA) functions as an extrinsic signal that regulates patterning of rhombomeres in the hindbrain and neuronal differentiation in the spinal cord [15]–[17], but the role of RA in forebrain development remains unclear. RA is derived from vitamin A through a two-step enzymatic process, employing retinol dehydrogenase (Rdh10) for oxidation of retinol to retinaldehyde, and retinaldehyde dehydrogenases Raldh1 (Aldh1a1), Raldh2 (Aldh1a2), and Raldh3 (Aldh1a3) for oxidation of retinaldehyde to RA, which then functions as a ligand for nuclear RA receptors [18]. A role for RA signaling during mouse striatal development is evident after E12.5 when *Raldh3*, expressed in the subventricular zone of the LGE [19], plays a required role in the up-regulation of dopamine receptor D2 expression [20]. Consistent with this finding, loss of RA receptor-beta (RARβ) in null mutant mice is associated with defects in striatal dopaminergic neurogenesis after E13.5 resulting in motor behavioral defects [21]. A recent study using *Rdh10* mutant embryos with reduced RA synthesis in the meninges suggested that RA is required for normal radial expansion of the dorsal cortex [22]. However, other studies have suggested that RA may not act in the embryonic cortex, as RA activity was detected in the LGE but not the cortex [23].

Here, we employ null mutants for *Raldh3* (*Aldh1a3*) and *Raldh2* (*Aldh1a2*) to ascertain the anatomical sites, cellular targets, and consequences of RA signaling in the embryonic forebrain. Our results provide evidence that *Raldh3* expression in the LGE is a major source of RA production in the embryonic forebrain and is required for GABAergic differentiation from LGE-derived progenitors in the basal ganglia. Furthermore, our findings suggest that RA generated in the meninges by *Raldh2* is not required to stimulate radial expansion of the cortex as previously suggested. We also report that RA induces GABAergic differentiation in neurons generated from LGE-derived neurospheres and human embryonic stem cells, thus implicating a role for RA as a GABAergic differentiation factor both in vivo and in vitro.

## Results

### RA Signaling in the Embryonic Forebrain

**Figure 1:**
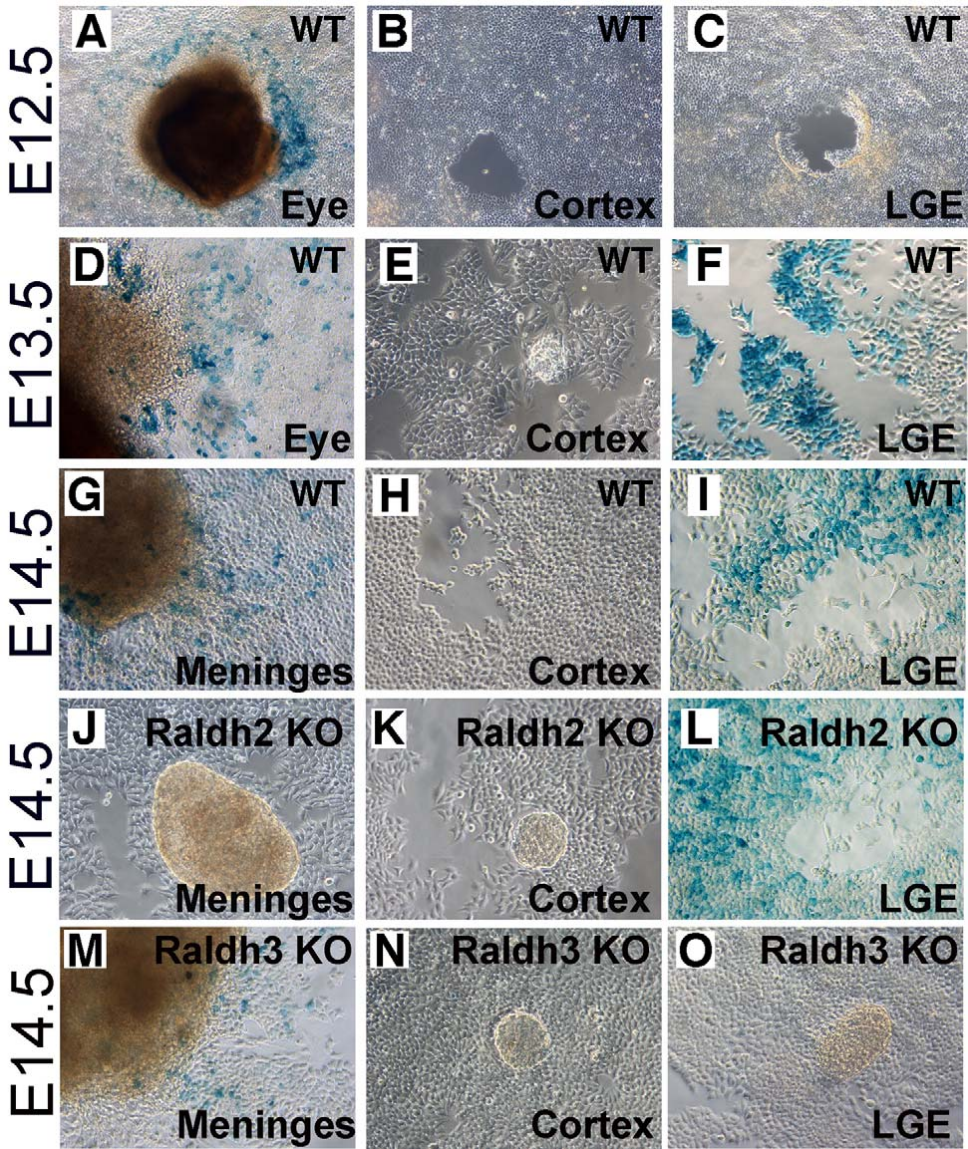
Endogenous RA activity in the developing forebrain. Tissues were cultured as explants on a monolayer of F9 *RARE-lacZ* RA-reporter cells, then stained for β-galactosidase activity. (A–C) At E12.5, cortex and LGE were both negative, whereas eye was positive. (D–F) At E13.5, LGE and eye explants induced RA activity in the reporter cells, but not cortex. (G–I) At E14.5, meninges and LGE induced RA activity while the reporter cells surrounding E14.5 cortical explants remained negative. (J–L) For E14.5 *Raldh2*−/− (KO) tissues, meninges and cortex were negative, while LGE explants remained positive. (M–O) For E14.5 *Raldh3*−/− (KO) tissues, meninges explants remained positive, while cortex and LGE were negative. For each genotype and stage, tissues from at least three embryos were analyzed with similar results.

Although *Raldh3* expression in the LGE from E12.5 to early postnatal stages suggests the LGE is a major site of RA action in the embryonic forebrain [19],[24], *Raldh2* and *Rdh10* are expressed in the meninges beginning at E12.5–E13.5, suggesting that RA synthesized there may regulate corticogenesis [22],[25]. In order to better define the timing and location of RA signaling in the developing forebrain from E12.5–E14.5, we employed a tissue explant RA reporter cell assay [26]. Cortex and LGE tissues were dissected from E12.5 to E14.5 embryos and grown as explants in co-culture with the RA-reporter cells. As positive controls, eye (E12.5 to E13.5), which expresses *Raldh1* and *Raldh3*, and meninges (E14.5), which expresses *Raldh2*, were dissected from the same embryos. Reporter cells co-cultured with cortex or LGE from E12.5 wild-type embryos displayed no RA activity, whereas eye explants did (Figure 1A–C); lack of RA activity in E12.5 LGE explants may be due to low initial *Raldh3* expression. In accordance with the increase in *Raldh3* expression in the LGE after E12.5 [19], LGE explants from both E13.5 and E14.5 induced strong RA activity in the surrounding reporter cells (Figure 1F,I). In contrast, E13.5 and E14.5 cortical explants remained unable to induce RA activity, whereas meninges and eye explants at these stages exhibited RA activity (Figure 1D,E,G,H). To verify that RA activity detected in the LGE is due to *Raldh3* expression, we found that loss of RA synthesis by *Raldh3* resulted in lack of RA activity in *Raldh3*−/− LGE explants but had no effect on RA activity in meninges (Figure 1M–O). Using an *Raldh2*−/− mutant model we found that all RA activity detectable in wild-type meninges at E14.5 was eliminated in *Raldh2*−/− meninges (Figure 1G,J). *Raldh2*−/− cortical explants contained no RA activity as observed in wild-type (Figure 1H,K), while RA activity was still observed in *Raldh2*−/− LGE (Figure 1I,L). Together, the above findings demonstrate that RA produced by *Raldh3* in the LGE can activate transcription in the basal ganglia, whereas RA produced by *Raldh2* in the meninges does not activate transcription in the adjacent cortex.

Exposure of the reporter cells to a range of RA concentrations between 1 nM and 1 µM provided a dose-response for RA activity (Figure S1); a concentration of 1 nM was sufficient to activate the reporter line as previously reported [26]. Thus, the fact that RA activity was not detected in cortical explants from E12.5 to E14.5 indicates that RA is present at very low levels in the cortex. However, recent studies proposed a role for RA in corticogenesis and additionally reported a value for the concentration of RA in the mouse E14.5 cortex (0.28 µmole/mg) [22], which is seven orders of magnitude higher than that previously reported for mouse E13.5 forebrain (12 pmol/g) [27]. The former value (presented as µmole/mg rather than pmol/g) is most likely in error as other studies reported RA concentrations in adult mouse cortex as 16 pmol/g and adult striatum as 78 pmol/g [28], but this leaves in doubt how much RA was actually detected in E14.5 cortex.

### LGE-Derived Progenitors Maintain Their RA Activity In Vitro

**Figure 2:**
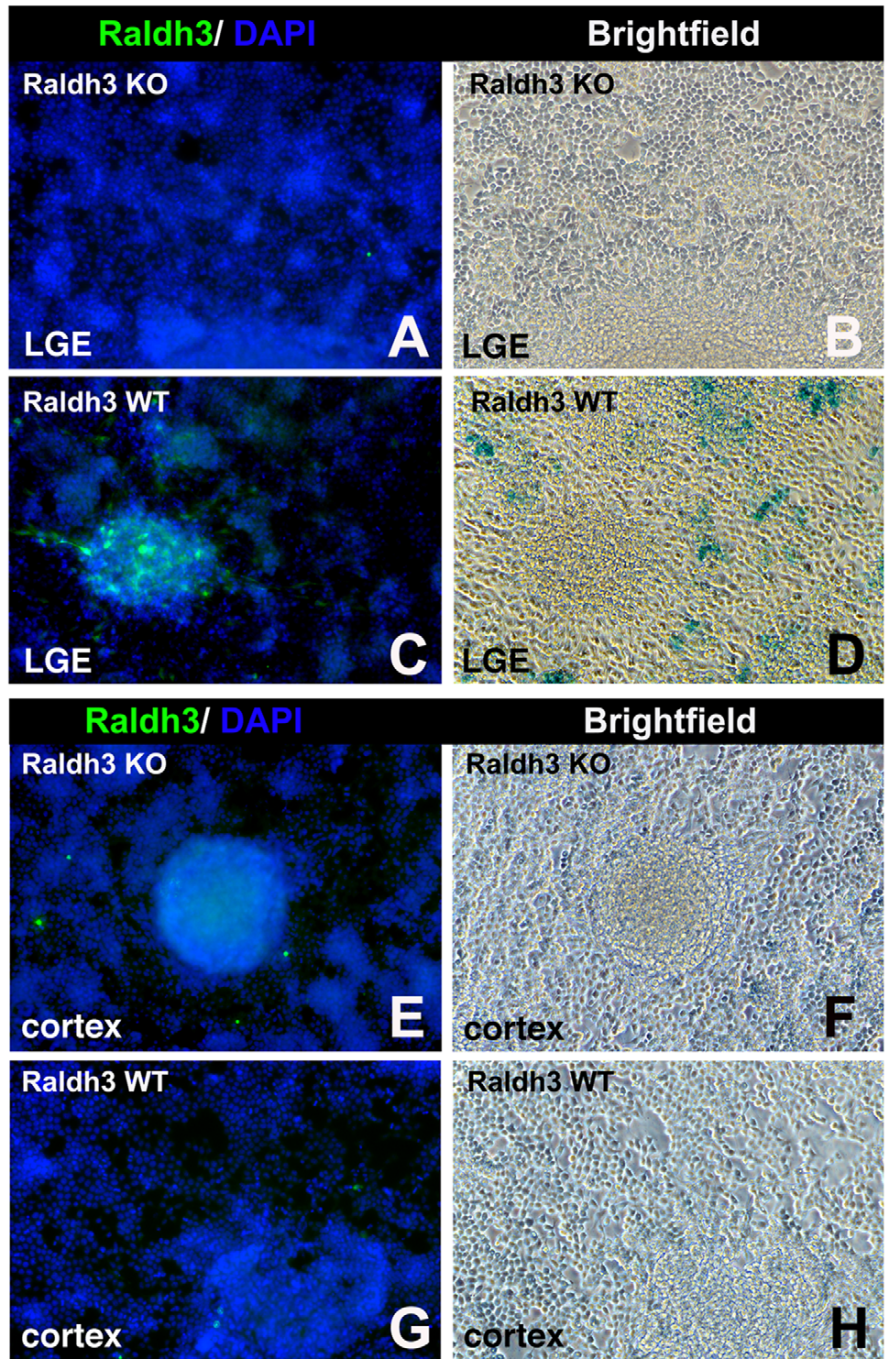
*Raldh3* is responsible for RA activity in neurospheres derived from the LGE but cortex-derived neurospheres lack RA activity. (A–D) Neurospheres generated from the LGE of E14.5 wild-type (WT) embryos exhibit Raldh3 immunoreactivity and they induce RA activity when co-cultured with F9-RARE-lacZ RA-reporter cells (*n* = 4); neurospheres derived from *Raldh3*−/− LGE always lacked both Raldh3 detection and RA activity (*n* = 4). (E–H) Neither Raldh3 nor RA activity were detected in cortex-derived neurospheres from either wild-type or *Raldh3*−/− (KO) embryos (*n* = 4).

Our observation that RA activity during forebrain development is due primarily to *Raldh3* expression in the LGE prompted us to investigate if neural precursors isolated from E14.5 LGE maintain their *Raldh3* expression and RA activity when expanded in vitro under mitogen stimulation. Hence, we employed immunocytochemistry with a Raldh3 antibody together with the tissue explant RA bioassay using neurospheres generated from E14.5 LGE and cortex of wild-type and *Raldh3*−/− embryos. RA activity and Raldh3 immunostaining were detected in neurospheres derived from wild-type E14.5 LGE (Figure 2C–D). In contrast, both Raldh3 immunostaining and RA activity were eliminated in LGE-derived neurospheres from *Raldh3*−/− embryos (Figure 2A–B). Neither Raldh3 immunostaining nor RA activity were found in neurospheres derived from the cortex of either wild-type or *Raldh3*−/− embryos (Figure 2E–H). These results further confirm that *Raldh3* expression in the LGE is responsible for RA synthesis and additionally showed that neurospheres expanded from LGE cells maintain their RA activity.

### GABAergic Differentiation of LGE-Derived Neurospheres Depends on RA

**Figure 3:**
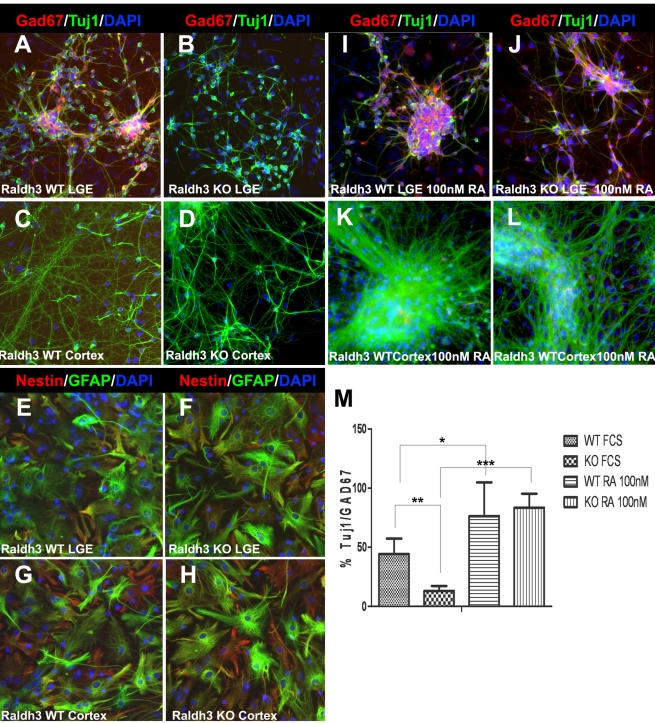
RA induces GABAergic differentiation in neurosphere-derived cells from the LGE. (A–B) Neurosphere-expanded wild-type (WT) cells derived from the E14.5 LGE give rise to Gad67-positive and Tuj1-positive cells after 1 wk of differentiation; under the same conditions no Gad67-positive cells were detected in differentiating cultures derived from *Raldh3*−/− (KO) LGEs. (C–D) Gad67 was not detected in differentiating cultures of E14.5 neurospheres from either wild-type or *Raldh3*−/− cortex. (E–H) Cells derived from E14.5 *Raldh3*−/− LGE and cortex neurospheres both express nestin and GFAP at indistinguishable levels from cells derived from wild-type neurospheres. (I–J) RA induces GABAergic differentiation of LGE-derived neurospheres; addition of 100 nM RA for 1 wk in the differentiation medium of expanded LGE cells resulted in an increase of Gad67+/Tuj1+ neurons in both wild-type and *Raldh3*−/− cultures. (K–L) Cortex-derived neurospheres treated with 100 nM RA exhibited no Gad67 detection. (M) Quantification of the percentage of LGE neurosphere-derived Tuj1-positive neurons also positive for Gad67 showed that in differentiating medium containing only fetal calf serum (FCS) with no added RA, the percentage of Gad67+/Tuj1+ cells derived from *Raldh3*−/− LGE neurospheres was significantly lower than that of wild-type LGE neurospheres. Following addition of 100 nM RA to the differentiation medium for 1 wk, the majority of Tuj1-positive neurons derived from both wild-type and *Raldh3*−/− LGE neurospheres were also Gad67-positive. The percentage was calculated by dividing the immunopositive cell number with the total number of DAPI-stained nuclei. Values are listed as mean ± SEM; * *p*<0.05; ** *p*<0.01; *** *p*<0.001.

We investigated whether RA has an effect on the differentiation potential of regionally derived neurosphere cultures. Neurospheres from LGE and cortex of E14.5 wild-type and *Raldh3*−/− embryos were differentiated for 7 d and subsequently analyzed immunocytochemically with antibodies against the pan-neuronal marker β-tubulin-III (Tuj1), the GABA-synthesizing enzyme glutamic acid decarboxylase (Gad67), the neural progenitor marker nestin, and the astrocytic marker glial fibrillary acidic protein (GFAP). Many wild-type LGE neurospheres untreated with RA were found to co-express Tuj1 and Gad67, indicating they have differentiated and matured into a GABAergic phenotype (44.3%±13.0%), however very few Tuj1/Gad67-positive cells were detected in differentiated cultures of *Raldh3*−/− LGE neurospheres (13.2%±4.1%) (Figure 3A–B,K). Tuj1-expressing neurons that differentiated from cortical *Raldh3*−/− neurospheres appeared to have a similar morphology to those generated from cortical wild-type neurospheres, and Gad67 was never detected (Figure 3C–D). Nestin-positive progenitors and cells with an astrocytic morphology expressing GFAP appeared similar in LGE and cortical differentiated cultures derived from wild-type and *Raldh3*−/− neurospheres (Figure 3E–H).

LGE neurospheres from both wild-type and *Raldh3*−/− embryos were differentiated in the presence of RA in order to further test the effect of RA on GABAergic neuronal differentiation. After 1 wk of differentiation in the presence of 100 nM RA, the majority of the generated neurons in *Raldh3*−/− and wild-type cultures were GABAergic, as observed by double staining for Tuj1 and Gad67 (Figure 3I–J). However, when cortical neurospheres were differentiated in the presence of RA, Gad67 was never detected in either wild-type or *Raldh3*−/− differentiating cultures (Figure 3K–L). Quantification of GABAergic neuron differentiation from wild-type and *Raldh3*−/− LGE neurospheres with or without added RA showed a significant increase of Gad67-positive neurons in the presence of RA. The proportion of GABAergic cells derived from *Raldh3*−/− LGE neurospheres increased significantly from 13.2%±4.1% under control conditions to 83.37%±11.75% when the neurospheres had been differentiated in 100 nM RA (Figure 3M). The proportion of GABAergic neurons in cultures of wild-type LGE neurospheres increased from 44.3%±13.0% under control conditions to 76.45%±11.75% after treatment with 100 nM RA (Figure 3M). Thus, E14.5 LGE derived cells from *Raldh3*−/− embryos can be expanded as neurospheres and are able to differentiate into neurons and glia, but they are unable to differentiate into GABAergic neurons unless RA is added.

### *Raldh3* Expression in Basal Ganglia Is Required for GABAergic Differentiation in the LGE

**Figure 4:**
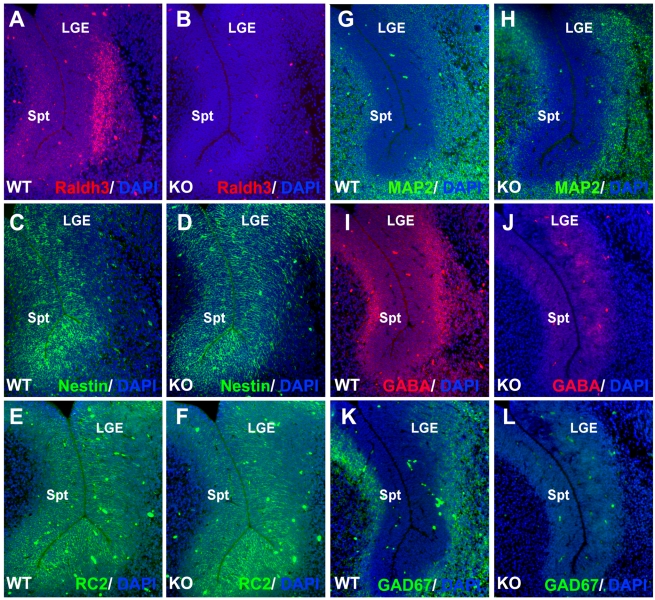
Loss of RA signaling creates a defect in GABAergic differentiation in the LGE. Immunofluorescence was performed on E14.5 forebrain coronal sections of wild-type (WT) and *Raldh3*−/− (KO) embryos. (A–B) Raldh3 immunoreactivity in the subventricular zone of the LGE is lost in the mutant. (C–H) Loss of Raldh3 does not affect detection of neural progenitor marker nestin, radial glial marker RC2, or neuronal marker MAP2. (I–L) Detection of both GABA and Gad67 is greatly reduced in the LGE and septum of *Raldh3*−/− embryos. Similar results were observed for all mutants analyzed (*n* = 3). LGE, lateral ganglionic eminence; Spt, septum.

The observation that RA generated by Raldh3 induces GABAergic differentiation of neural precursors in vitro prompted us to investigate if RA signaling is required for GABAergic differentiation in the developing forebrain. We examined a panel of markers for both neural progenitors and differentiated neurons in forebrains from E14.5 wild-type and *Raldh3*−/− embryos. Raldh3 protein was observed at high levels in the SVZ of the LGE; Raldh3 was not detected in the *Raldh3*−/− forebrain (Figure 4A–B). Nestin and RC2 immunoreactivity was not significantly changed in *Raldh3*−/− versus wild-type basal ganglia at E14.5, suggesting that generation of neural progenitors is not affected when RA signaling is disrupted (Figure 4C–F). In order to determine the proliferative capacity of these neural progenitors, double immunohistochemistry was performed with the proliferation marker Ki67 and the radial glia/progenitor marker nestin (Figure S2A–B). We observed no reduction in the number of LGE proliferating progenitors in *Raldh3*−/− embryos compared to control embryos, showing that both generation and proliferation of neural progenitors is not affected when RA signaling is disrupted (Figure S2C).

MAP2 immunostaining marking postmitotic neurons [29] was unaffected in *Raldh3*−/− basal ganglia (Figure 4G–H). The numbers of MAP2-expressing neurons were quantified in the striatum, cortex, and septum from wild-type and *Raldh3*−/− embryos. MAP2-expressing cells did not change in number in these regions of the forebrain in *Raldh3*−/− embryos, confirming that neurogenesis was not affected (Figure S2D).

A defect in GABAergic differentiation was observed when RA signaling is lost in the basal ganglia. Gad67-positive cells are normally present along the SVZ from the LGE to the septum (Figure 4K), and the pattern of GABA immunoreactivity is normally similar to Gad67 although GABA-positive cells extend into the ventricular zone (Figure 4I); this is probably due to the fact that while Gad67 immunoreactivity marks only cells with GABA production (i.e. GABAergic cells), GABA immunoreactivity could mark cells synthesizing GABA plus cells that uptake GABA released by Gad67-positive cells. In *Raldh3*−/− embryos, detection of both Gad67 and GABA was nearly eliminated in the LGE and septum (Figure 4J,L). At E12.5, when *Raldh3* expression in the LGE has just initiated, we observed that GABA was detected in the MGE and LGE in a pattern that was not significantly different between wild-type and *Raldh3*−/− forebrain; GABA detection in the LGE at E12.5 was at a lower level than that seen at E14.5 (Figure S3A–B). As RA activity is not yet detected in the LGE at E12.5 but is seen by E13.5 (Figure 1C,F), these findings demonstrate that RA signaling initiating after E12.5 is required to stimulate the high level of GABAergic differentiation normally observed in the LGE by E14.5.

The cellular source of RA in the LGE has previously been associated with newly born neurons in the subventricular zone expressing *Raldh3* [24]. We analyzed Raldh3 immunoreactivity in two distinct types of cells, radial glia and postmitotic neurons of the LGE at E14.5. Double-labeling studies demonstrated that none of the RC2-positive radial glia exhibit Raldh3 detection, although the radial processes of these cells were observed next to Raldh3-positive cells localized in the SVZ that did not possess radial processes (Figure S4A–C). In contrast, most Raldh3-expressing cells were also labeled with neuronal marker MAP2 (Figure S4D–F). The above results provide further evidence that Raldh3-expressing cells in the LGE are newly born neurons defining a discrete region of the SVZ.

### RA Is Required to Stimulate GABAergic Differentiation in the Striatum

**Figure 5:**
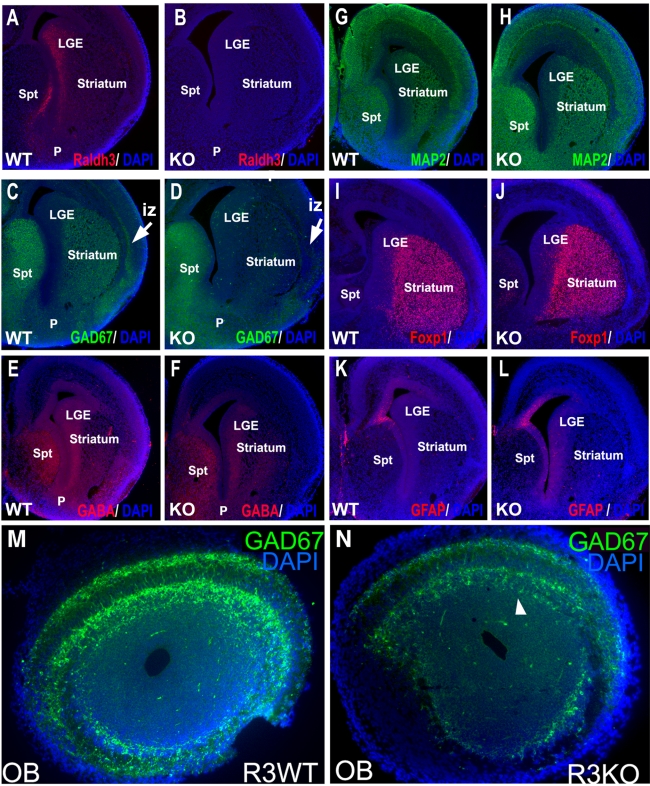
RA is required for GABAergic differentiation of striatal projection neurons and interneurons migrating to the cortex and olfactory bulb. Immunofluorescence was performed on E18.5 forebrain coronal sections of wild-type (WT) and *Raldh3*−/− (KO) embryos. (A–B) Raldh3 immunoreactivity in the subventricular zone of the LGE and septum is lost in the mutant. (C–F) In *Raldh3*−/− embryos, detection of both Gad67 and GABA is greatly reduced in the LGE, striatum, and cortex (note arrows for Gad67); septum is unaffected. (G–L) Loss of Raldh3 does not affect detection of neuronal marker MAP2, striatal projection marker Foxp1, or astrocytic marker GFAP. (M–N) *Raldh3*−/− embryos exhibit a reduction of Gad67-positive neurons migrating from the LGE to the olfactory bulb when compared to wild-type. All mutants analyzed generated similar results (*n* = 3). iz, intermediate zone; LGE, lateral ganglionic eminence; OB, olfactory bulb; P, pallidum; Spt, septum.

In E18.5 wild-type embryos, Raldh3 detection remains strongest along the SVZ of the LGE (particularly high in the dorsal LGE) with weaker detection further ventrally along the septum; Raldh3 immunoreactivity was eliminated in *Raldh3*−/− forebrain (Figure 5A–B). As observed at E14.5, MAP2 immunostaining was unaffected in *Raldh3*−/− versus wild-type forebrain at 18.5 (Figure 5G–H). At E18.5, GABAergic differentiation in the striatum was nearly eliminated in *Raldh3*−/− forebrain as monitored by Gad67 immunoreactivity (Figure 5C–D); GABA detection was reduced in striatum but less so than Gad67 possibly due to diffusion of GABA still generated ventral of the striatum (Figure 5E–F). Thus, not all regions of the basal ganglia were affected by the disruption of RA signaling, as both Gad67 and GABA immunoreactivity appear relatively normal in the pallidum and septum at E18.5 (Figure 5C–F).

The subcortical telencephalon is known to be the source of GABAergic projection neurons that migrate radially from the ventricular progenitor zone to reach their final destination. The LGE gives rise to GABAergic striatal projection neurons [1],[30]–[35], while the MGE gives rise to GABAergic projection neurons of the pallidum, septum, and nucleus basalis [1],[30]. In order to determine if differentiation of striatal projection neurons is affected in E18.5 *Raldh3*−/− embryos, we examined Foxp1, a marker for these neurons [36], and found that *Raldh3*−/− embryos display normal Foxp1 immunoreactivity in the striatum (Figure 5I–J). This observation supports our previous findings demonstrating that *Raldh3* is not required to generate DARPP32-positive neurons, another marker of striatal medium-sized spiny projection neurons [20]. Instead, our findings demonstrate that RA is required for striatal projection neurons to acquire a GABAergic fate.

Our results indicate that RA is required to stimulate GABA synthesis in LGE-derived progenitors. To characterize other aspects of the GABAergic phenotype in *Raldh3*−/− mutants, we analyzed expression of the vesicular GABA transporter (VGAT, Viaat), a transporter that mediates accumulation of GABA into the synaptic vesicles before exocytotic release to the synaptic cleft [37]. Expression of VGAT appeared normal in *Raldh3*−/− forebrain (Figure S5A–B), indicating that RA is not required for this aspect of GABAergic differentiation.

Next, we wanted to investigate whether disruption of RA signaling could induce defects in the specification of other neuronal populations. Previous studies have shown that loss of *Raldh3* or RARβ in the striatum results in down-regulation of dopamine receptor D2 in the nucleus accumbens [20],[21]. However, in *Raldh3*−/− forebrain no difference was found in the expression of tyrosine hydroxylase (TH), a marker of dopaminergic neurons (Figure S5C–D). Also, we observed no difference in vesicular glutamate transporter (VGLUT), a marker of glutamatergic neurons (Figure S5E–F).

In addition to neuronal markers, we examined whether loss of RA affects glia. We showed above that radial glia differentiation is not affected by loss of *Raldh3* (Figure 4E–F). We also analyzed astrocyte differentiation by analyzing expression of the astrocytic marker GFAP. We did not detect any difference in GFAP immunoreactivity in E18.5 *Raldh3*−/− forebrain compared to wild-type controls (Figure 5K–L). Our findings thus suggest that RA is not required for gliogenesis or generation of radial projection neurons.

Taken together, our observations at E12.5–E18.5 demonstrate that RA is required to stimulate a high level of GABAergic differentiation first in the LGE and then later in the striatum but that a Raldh3-independent mechanism for GABAergic differentiation occurs in the MGE/pallidum and septum.

### RA Is Required for Differentiation of LGE-Derived GABAergic Interneurons Migrating to the Cortex and Olfactory Bulb

In addition to GABAergic projection neurons, progenitor cells in the LGE produce GABAergic interneurons that migrate tangentially mostly within the cortical intermediate zone, whereas GABAergic interneurons migrating from the MGE disperse into the cortical plate [5],[38]–[40]. Also, cells derived from the dorsal SVZ of the LGE generate many olfactory bulb interneurons via a rostral migratory pathway [40]–[42]. At E18.5, Gad67 immunoreactivity normally extends from the striatum into the intermediate zone of the cortex marking a population of LGE-derived interneurons, but this zone of Gad67 detection was markedly reduced in *Raldh3*−/− cortex (Figure 5C–D). Dlx2 is an early marker of GABAergic progenitors in the basal ganglia that is required for GABAergic interneuron migration to the cortex [14]. Dlx2 immunoreactivity was not changed in *Raldh3*−/− forebrain from E12.5–E18.5, demonstrating that interneurons are generated in the basal ganglia and migrate to the cortex in the absence of RA signaling (Figure S3C–H). Additionally, detection of Gad67 in the *Raldh3*−/− olfactory bulb was also clearly reduced compared to wild-type (Figure 5M–N). Apart from the dorsal LGE, recent studies have shown that additional telencephalic areas may also contribute to olfactory bulb interneurons [43]–[45], which may explain our observed partial elimination of Gad67 immunoreactivity in the *Raldh3*−/− olfactory bulb. The above findings provide evidence that RA synthesis controlled by *Raldh3* is required for GABAergic differentiation of interneurons that originate from progenitors in the LGE then migrate tangentially to the cortex and olfactory bulb.

### RA Stimulates GABAergic Differentiation of Human Embryonic Stem Cells

**Figure 6:**
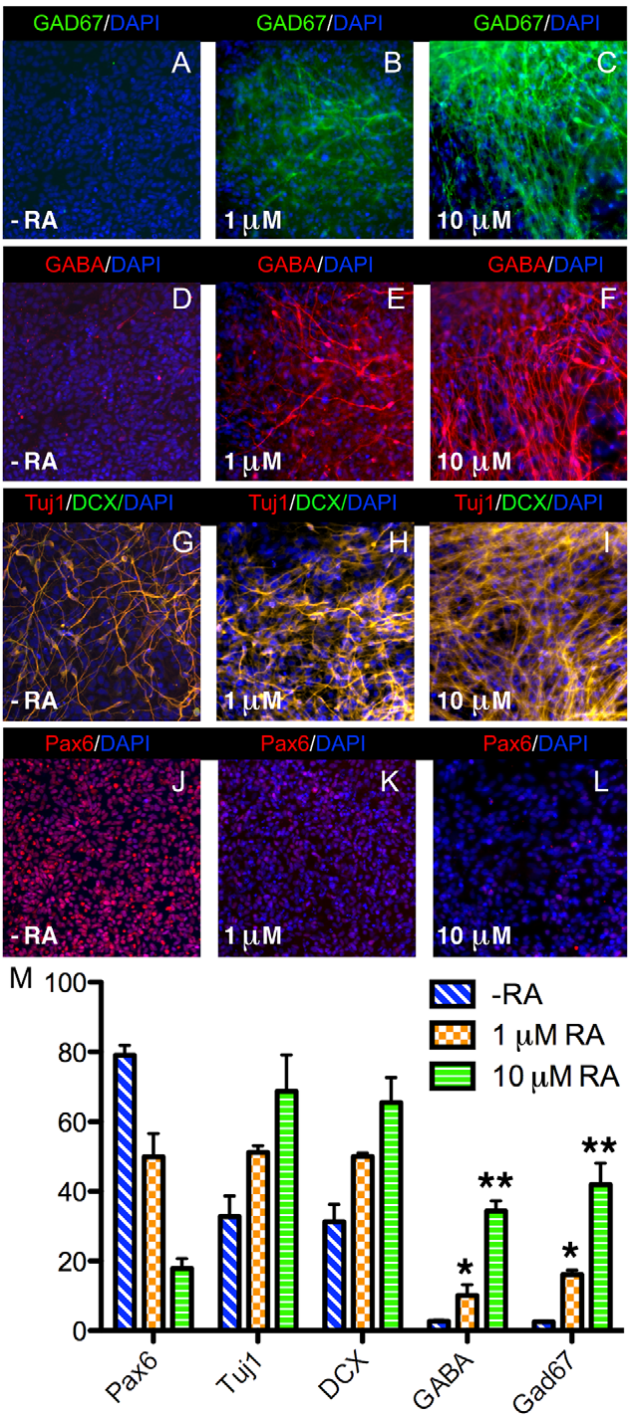
RA induces GABAergic differentiation of human embryonic stem cells. Embryoid bodies derived from H9 human embryonic stem cells were treated for 3 d with vehicle, 1 µM RA, or 10 µM RA; then neural rosettes differentiating from these cultures were analyzed immunocytochemically 18 d after RA treatment ended. (A–F) RA treatment increased the number of neurons positive for Gad67 and GABA. (G–I) Tuj1 and DCX double-staining shows that RA treatment increases the number of Tuj1-positive neurons, and essentially all are also positive for DCX, which marks migrating neurons. (J–L) RA treatment decreased the number of neurons positive for the neural progenitor marker Pax6. (M) Quantitative analysis of various cell types in differentiating cultures showed that 1 µM RA or 10 µM RA significantly induced the GABAergic neuron phenotype (Gad67-positive) with a concomitant decrease of Pax6-expressing neural progenitors. Percentages were calculated by dividing the immunopositive cell number with the total number of DAPI-stained nuclei. Data are presented as mean ± SEM; * *p*<0.01 and ** *p*<0.001 (untreated versus treated).

We investigated whether our findings may be useful to generate GABAergic neurons from human embryonic stem (ES) cells for potential cell replacement therapies. Following RA treatment of embryoid bodies and propagation of neural rosettes, cultures were processed immunocytochemically with antibodies against Pax6 (a marker for neural progenitors), Doublecortin (DCX; a marker for immature migrating neurons), the pan-neuronal marker Tuj1, plus GABA and Gad67. With no RA added in the differentiation medium a large proportion of the cells were Pax6-positive (79.9%±5.01%), indicating they were neural progenitors (Figure 6J,M), and many cells exhibiting neuronal processes were Tuj1-positive (32.8%±10.3%) and colocalized with DCX (31.3%±8.6%), suggesting they were immature neurons (Figure 6G,M). However, very few Gad67 (2.7%±0.6%) positive cells were detected in cultures with no RA added, suggesting that GABAergic differentiation is not favored under these conditions (Figure 6A,D). Treatment of embryoid bodies with 1 µM RA resulted in a significant increase in both the number of Tuji1/DCX+ neurons that were forming extensive neuronal networks and GABAergic neurons detected with Gad67 (16.2%±2.1%) (Figure 6B,E,M). Moreover, the proportion of Pax6-positive cells was reduced to almost half with 1 µM RA (49.9%±11.6%), suggesting that more progenitor cells had differentiated to immature migrating neurons co-expressing Tuj1 and DCX (Figure 6H,K,M). Addition of 10 µM RA further increased the percentage of GABAergic neurons positive for Gad67 (41.9%±10.7%) (Figure 6C,F,M) and further decreased the number of Pax6-positive progenitors (17.9%±4.9%) (Figure 6L,M). Under these differentiation conditions, RA was able to drive GABAergic differentiation in almost half of the cells generated (Figure 6M), suggesting that RA treatment is quite useful for induction of GABAergic differentiation in vitro.

To gain further insight on the subtype identity of the RA-induced GABAergic neurons generated in our cultures, we examined expression of region-specific transcription factors previously associated with the specification of both GABAergic interneurons and projection neurons. Many Tuj1-positive cells were also immunopositive for Dlx2, which is expressed in GABAergic precursors in the basal ganglia of the telencephalon, differentiating into both interneurons and striatal projection neurons (Figure S6A). However, no Tuj1-positive cells were found to be positive for Foxp1, a striatal projection neuron marker (Figure S6B). Lim1/2 is a LIM homeodomain protein marking interneurons of the diencephalon and spinal cord [17],[46]–[48]. Many GABA-positive neurons also expressed Lim1/2, suggesting they acquire a GABAergic interneuron phenotype (Figure S6C). Islet1 (Isl1), another LIM homeodomain protein, is expressed in the ventral forebrain where it marks telencephalic GABAergic projection neurons; Isl1 also marks diencephalic interneurons when co-expressed with Lim1/2 and GABA [46],[48]. No cells in our culture co-expressed GABA and Isl1, further suggesting that GABA-positive neurons in our cultures do not acquire striatal projection neuron or diencephalic interneuron identities (Figure S6D). We found that 43.7%±11.94% of GABA-positive cells expressed Lim1/2 and 37.5%±11.5% expressed Dlx2 (Figure S6E). Together, these data show that our human ES cell differentiation protocol induces a heterogeneous population of GABA-positive interneurons acquiring either telencephalic or spinal cord identities, but that it does not favor generation of GABA-positive striatal projection neurons.

### RA Is Not Required for Radial Expansion of the Embryonic Cortex

**Figure 7:**
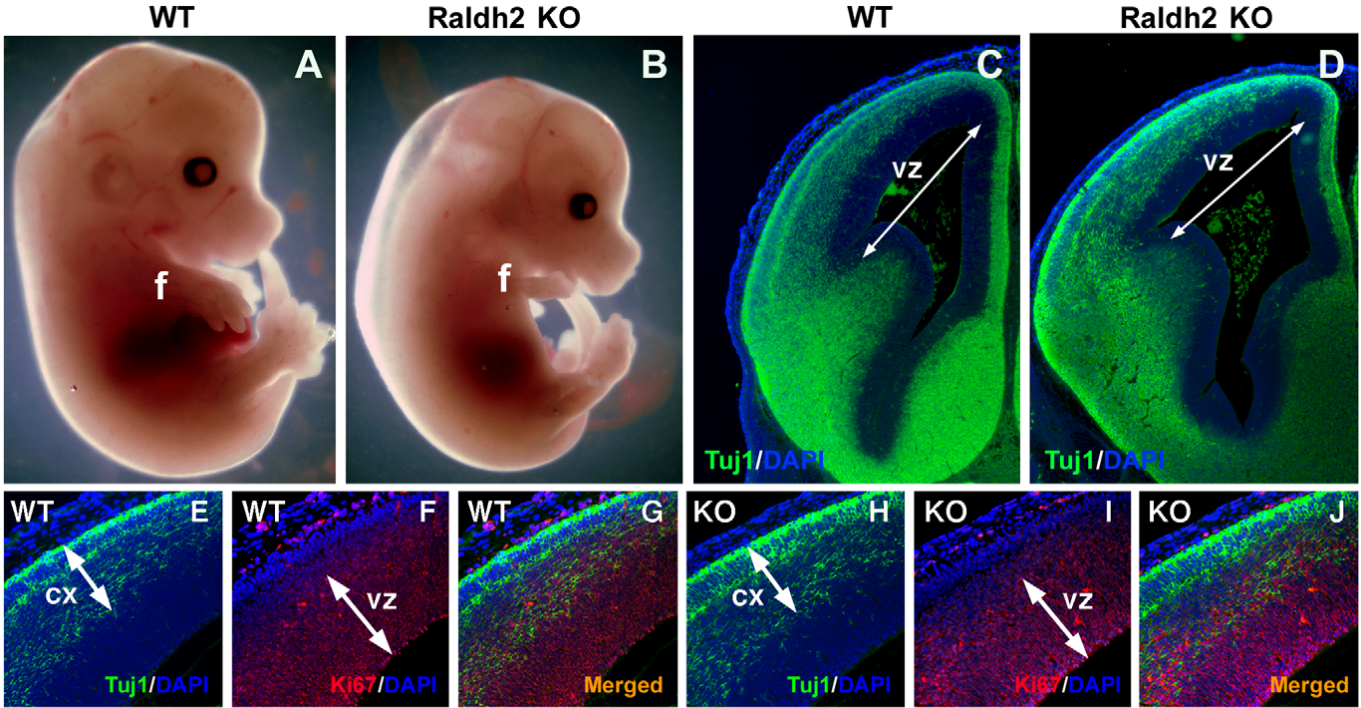
Cortical expansion appears normal in embryos lacking RA synthesis in meninges. (A–B) *Raldh2*−/− embryos at E14.5 exhibit relatively normal craniofacial development while they display stunted forelimbs (f). (C–D) Tuj1 labeling of postmitotic neuronal layer in E14.5 wild-type (WT) and *Raldh2*−/− (KO) forebrains. Arrows (same length) demonstrate that *Raldh2*−/− mutant does not exhibit a change in medial-lateral width of the dorsal ventricular zone (vz; labeled with DAPI) compared to wild-type. (E–J) Double-labeling of E14.5 forebrains with Tuj1 (green) and Ki67 (red; proliferative progenitor layer). Arrows (same length) demonstrate that the *Raldh2*−/− forebrain exhibits no change in radial expansion of the cortex (cx) or ventricular zone (vz) compared to wild-type. Similar results were observed for all mutants analyzed (*n* = 3).

Previous studies using an *Rdh10* ethylnitrosourea (ENU) mutant, which reduces retinaldehyde needed for Raldh2 to catalyze RA synthesis in the meninges, suggested that this source of RA is required for radial expansion of the cortex; a reduction in radial expansion of the cortical postmitotic neuronal layer was proposed to result in a concomitant lateral increase in the proliferative progenitor population in the ventricular zone [22]. *Raldh2*−/− embryos, which completely lack meninges RA activity (Figure 1G,J), present an excellent model to examine whether RA is required for corticogenesis since Raldh2 catalyzes the final step of RA synthesis in the meninges. At E14.5, the head region of *Raldh2*−/− embryos appeared to have developed relatively normally while they invariably displayed stunted forelimbs (Figure 7A–B), which we have previously shown is due to a lack of RA synthesis by Raldh2 in trunk mesoderm [49],[50]. Thus, *Raldh2*−/− mutants do not exhibit massive head deformities like those reported for *Rdh10* mutants [51]. We analyzed expression of Tuj1 and the proliferative marker Ki67 in coronal brain sections of both wild-type and *Raldh2*−/− embryos at E14.5. The medial-lateral length of the ventricular zone in the dorsal forebrain of the *Raldh2*−/− mutant appeared similar to that of the wild-type embryo (Figure 7C–D). Moreover, double immunostaining for Tuj1 and Ki67 revealed no changes in radial expansion of the postmitotic Tuj1-expressing cortical layer nor the Ki67 proliferative zone in the *Raldh2*−/− cortex when compared to wild-type (Figure 7E–J). Examination of MAP2, another marker for postmitotic neurons, also demonstrated no difference in medial-lateral length for the mutant dorsal ventricular zone (Figure S7A–B) and no difference in radial width for the mutant cortex (Figure S7C–D). Finally, examination of RC2, a marker of radial glia whose somata reside in the ventricular zone of the cortex and whose radial processes span the entire distance to the pial surface, showed no difference between the *Raldh2*−/− and wild-type cortex (Figure S7E–F). The fact that *Raldh2*−/− embryos retain a normal ratio of cortical progenitors to postmitotic neurons with no apparent morphological defects, in conjunction with a complete lack of RA activity in mutant meninges and cortical explants, suggests that RA is not required for embryonic corticogenesis. The contradiction between our results with *Raldh2*−/− embryos and the results of others with *Rdh10* mutants [22] may be explained by the observation that *Rdh10* mutants, unlike *Raldh2*−/− embryos, exhibit severe craniofacial defects that distort the cranium and forebrain possibly resulting in a thinner cortex [51].

### Expression of Retinoic Acid Receptors in the Developing Forebrain

Previous studies have shown that RARα and RARβ are expressed during mouse forebrain development, whereas RARγ is undetectable [52],[53]; RARα was reported to be widespread in the embryonic forebrain, while RARβ was detected primarily in the striatum and is induced by RA. We examined expression of RARα and RARβ in E18.5 wild-type forebrains by in situ hybridization. RARα mRNA was widespread in the E18.5 forebrain including both the striatum and cortex, but expression was low or undetectable in the ventricular zone (Figure S8A). RARβ mRNA was detected in the striatum but not in the cortex or ventricular zone (Figure S8B). Taken together with our observation that the LGE/striatum is a major localized site of RA synthesis during forebrain development due to *Raldh3* expression, overlapping expression of both RARα and RARβ in the striatum further suggests that this is a major site of local RA-mediated induction and signaling.

## Discussion

In this study we demonstrate a novel requirement of RA generated by *Raldh3* for GABAergic differentiation in the basal ganglia. In contrast, RA activity is not detected in the cortex at any stages examined despite detection of RA activity in the adjacent meninges, which requires *Raldh2*. Even if the cortex does receive a small amount of RA from the meninges that we cannot detect, our findings with *Raldh2*−/− embryos lacking RA activity in the meninges demonstrate that this source of RA is unnecessary for cortical expansion as suggested by a recent study [22]. Thus, unlike the cortex, the LGE represents an unambiguous site of RA action during forebrain development, and loss of RA in the LGE results in a loss of GABAergic differentiation.

Our studies revealed that at E12.5, when *Raldh3* expression is barely detectable in the LGE and RA activity is not yet detected, *Raldh3*−/− embryos maintain expression of the regulatory gene *Dlx2* and early aspects of GABAergic differentiation in the progenitor domains of the basal ganglia. However, by E14.5, when *Raldh3* expression has intensified in the LGE and RA activity is easily detectable, RA generated by Raldh3 is required to stimulate GABAergic differentiation in the LGE by inducing Gad67 needed for GABA synthesis. At E18.5, Raldh3 is required to maintain GABAergic differentiation in the LGE, whereas a Raldh3-independent mechanism controls GABA synthesis in the MGE and septum. This observation suggests that the LGE is the main site of RA action along the SVZ. We observed that *Raldh3* expression in newly generated neurons at the border of the proliferative and postmitotic zones in the LGE coincides with a region that generates both GABAergic striatal projection neurons and GABAergic interneurons [5],[31]–[34],[40]. Our studies in *Raldh3*−/− embryos revealed that RA signaling stimulates a GABAergic phenotype in LGE-derived interneurons migrating to the olfactory bulb and cortex and that RA is required for Foxp1-positive striatal projection neurons to further differentiate to a GABAergic fate. As Dlx2 and VGAT were still expressed normally in the absence of RA, the role of RA in GABAergic differentiation may be limited to stimulation of Gad67 activity in the LGE to promote GABA synthesis.

As the appearance of GABAergic interneurons in the olfactory bulb and cortex is reduced rather than eliminated, our findings suggest that interneurons can still migrate to these locations in the absence of RA but that less interneurons have matured to a GABAergic phenotype. Other studies have shown that *Gsx2* (*Gsh2*), a homeobox gene specifying ventral character in the forebrain, is required for *Raldh3* expression in the LGE [54] and that *Gsx2* is required for specification of GABAergic interneurons that migrate from the LGE to the olfactory bulb [13]. In addition, differentiation of DARPP-32 striatal projection neurons is greatly reduced in *Gsx2* null embryos but not in conditional *Gsx2* mutants when *Gsx2* is progressively inactivated from E10.5–E18.5 [13] and also not in *Raldh3*−/− mutants [20]. Thus, early expression of *Gsx2* is required for correct DARPP-32 striatal projection neuron development, a time when there is no *Raldh3* expression in the forebrain. Taking into consideration the above, one can conclude that RA signaling exerts a specific role in specifying the GABAergic phenotype both for production of GABAergic interneurons and for further differentiation of striatal projection neurons to a GABAergic fate. Examination of the *Gad67* promoter proximal region revealed no evidence of a canonical RA response element (unpublished data), suggesting that *Gad67* may be an indirect target or may be controlled post-transcriptionally by RA signaling in the basal ganglia during GABAergic differentiation. As it is clear that RARα and RARβ are both expressed in the basal ganglia, null mutants or antagonists for these RA receptors may be useful to further examine the mechanism through which RA functions during stimulation of GABAergic differentiation. Further, as we show that endogenous RA signaling is preserved in primary LGE neurosphere cultures and is required to generate GABAergic neurons in vitro, such cells may prove useful in studying the mechanism of RA action during GABAergic differentiation.

A previous study suggested that *Foxc1* mutants fail to form a complete forebrain meninges and exhibit increased lateral expansion of the cortical ventricular zone and reduced neurogenic radial expansion due to the loss of RA produced by *Rdh10* and *Raldh2* in the meninges [22]. The major conclusions of that study were drawn by comparison of the cortical phenotype of the *Foxc1* mutants with that of an *Rdh10* ENU mutant [22],[55]. However, our studies on *Raldh2*−/− embryos lacking RA activity in the meninges demonstrate that RA is not required for radial expansion of the embryonic cortex. Additionally, RA receptors were not detected in the ventricular zone of the developing cortex, where RA was proposed to be required to induce neurogenic division of cortical progenitors. Together, these findings suggest that the dorsal forebrain phenotype in *Foxc1* mutants is RA-independent. The *Rdh10* ENU mutant employed for those forebrain studies [22] exhibits a very similar phenotype to another published *Rdh10* ENU mutant, which has severe neural crest-derived craniofacial defects that are responsible for distortion of the cranium as well as forebrain [51]. Thus, reduced radial expansion of the cortex and increased lateral expansion of the ventricular progenitor zone reported for *Rdh10* mutants [22] may not be due to a specific effect of RA on corticogenesis but rather a defect in cranial neural crest migration and differentiation that leads to the altered cortical morphology. Indeed, *Rdh10* mutants lack all RA activity in the head during the time when cranial neural crest is migrating due to loss of all retinaldehyde synthesis [51], whereas *Raldh2*−/− embryos still retain most cranial RA synthesis during this time due to expression of *Raldh1* and *Raldh3* in ocular and olfactory tissues [56]. Thus, head development in *Raldh2*−/− embryos is not grossly altered, allowing us to conclude that a lack of cranial RA activity specifically in the meninges does not lead to a defect in radial expansion of the cortex. Furthermore, *Raldh2* is not expressed in the dorsal meninges until E12.5 [22] or E13.5 [25], while the lengthening of the dorsal forebrain in *Foxc1* mutants is already evident at E12.5 [22]. Based on the above, it seems unlikely that RA produced and secreted in the dorsal meninges could be the neurogenic factor inducing the switch from symmetric to asymmetric division in the ventricular zone to affect embryonic cortical expansion. Alternatively, RA generated in the meninges by *Rdh10* and *Raldh2* might have another function. RA could diffuse in the opposite direction and control development of the skull, which is populated by cranial neural crest cells. Interestingly, a recent study showed that ablation of all three RA receptors (RAR alpha, beta, and gamma) in cranial neural crest cells results in agenesis or malformations of most of the craniofacial skeletal elements including the frontal and parietal bones, which are adjacent to the dorsal meninges [57]. Additionally, *Foxc1* hypomorphic mutants also exhibit malformation of the frontal bone [58], providing further evidence that RA generated in the meninges downstream of *Foxc1* may function in cranial neural crest differentiation.

RA treatment is known to facilitate terminal differentiation of neural progenitors derived from ES cells [48],[59]–[63]. Here we demonstrated that exposure of human ES-derived embryoid bodies to high concentrations of RA promotes differentiation of neuronal precursors to a high percentage of immature GABAergic neurons. Interestingly, although a low endogenous concentration of RA is sufficient to stimulate GABAergic differentiation of cells in the LGE at E14.5, a high concentration of RA is needed for GABAergic differentiation in embryoid bodies derived from ES cells. This may be due to the much more primitive nature of cells in an embryoid body (similar to cells in an early gastrula) compared to neuroepithelial cells of the late embryonic forebrain. As RA binds directly to DNA-bound nuclear receptors that interact with co-repressors and co-activators, we suggest that high concentrations of RA may exert tremendous epigenetic effects on embryoid body cells, driving them to both a neuronal and GABAergic fate. In addition, our RA treatment protocol generated GABAergic neurons exhibiting expression of interneuron transcription factors of either anterior (forebrain) or posterior (spinal cord) identity, but not striatal projection neuron identity. A previous study proposed that mouse ES cells differentiating in medium without RA acquired a GABAergic identity of ventral forebrain co-expressing Gad67 and Isl1 (most likely striatal projection neurons), while exposure to RA resulted in acquisition of a spinal cord interneuron identity [48]; those studies differed from ours in that RA treatment occurred at a later window during embryoid body formation and a lower concentration of RA was used. Thus, differences in the effects of RA on GABAergic interneuron identity in various culture systems may be dependent upon the timing and concentration of RA used.

Production of the inhibitory neurotransmitter GABA in the central nervous system depends on local neurons, and disturbed GABAergic neuron function has been associated with numerous neurological disorders including Huntington's disease, autism, schizophrenia, bipolar depression, and epilepsy [9]–[12]. GABAergic interneurons are a particularly attractive cell population for cell-based therapies of these disorders due to their ability to migrate, differentiate, and function following transplantation [64]–[66]. GABAergic interneuron precursors derived from mouse ES cells were shown to migrate, survive for several months, and exhibit neurochemical and electrophysiological characteristics of mature interneurons when transplanted into postnatal cortex [67]. Additionally, transplantation of GABAergic interneuron precursors reduced the number of seizures in epileptic mice [68]. Thus, generation of GABAergic interneurons from RA-treated human ES cells as we report here coupled with isolation of cells with forebrain character may provide useful candidate cells in cell replacement therapies for one or more of these neurological conditions.

## Materials and Methods

### RA-Deficient Mouse Models

*Raldh3*−/− embryos exhibiting postnatal lethality just after birth were previously described [20]. *Raldh2*−/− embryos exhibiting midgestation lethality have been described previously [69]. To prevent *Raldh2*−/− early lethality, the maternal diet was supplemented for a short time with a very low dose of RA as described previously [50]. Briefly, 0.1 mg of all-*trans*-RA (Sigma Chemical Co.) was added per gram of standard mouse chow and provided fresh to pregnant females at E6.75–E8.75. At E9.25 mice were returned to standard chow until embryos were collected at E14.5; *Raldh2*−/− mutants obtained with this method invariably exhibit stunted forelimbs and interdigital defects, demonstrating a loss of RA function in regions where *Raldh2* is responsible for RA synthesis [50]. Dietary supplementation with this dose of RA is indeed low as HPLC measurements have shown that it provides less RA to embryos than Raldh2 normally generates [70]. Administered RA is cleared within 12–24 h after treatment ends [69], thus allowing one to examine embryos at E10.5–E14.5 that now lack RA activity normally generated by Raldh2. Embryos were genotyped by PCR analysis of yolk sac DNA and were staged by designating noon on the day of the vaginal plug as E0.5. All mouse studies conformed to the regulatory standards adopted by the Animal Research Committee at the Sanford-Burnham Medical Research Institute.

### Neurosphere Preparation and Differentiation

Lateral ganglionic eminence (LGE) and cortex were dissected from E14.5 wild-type and *Raldh3*−/− mutant embryos and incubated in DMEM containing 0.1% trypsin and 0.05% DNase for 15 min at 37°C followed by mechanical dissociation. The cells were spun down and resuspended at a concentration of 100,000 cells/ml in basic medium DMEM-F12 supplemented with B27, 10 ng/ml basic fibroblast growth (bFGF) factor, and 20 ng/ml epidermal growth factor (EGF). No apparent differences in growth rate or appearance were observed for wild-type compared to *Raldh3*−/− neurosphere cultures. Neurospheres were differentiated by culturing on plates pre-coated with poly-L-ornithine. EGF and bFGF were removed from the expansion medium and 1% serum was added (differentiation medium). The spheres were maintained under differentiation conditions for 7 d in the presence or absence of 100 nM RA before fixation.

### Explant Culture and RA Bioassay

Tissue explants or neurospheres from wild-type and mutant embryos were cultured overnight on Sil-15 F9-RARE-lacZ RA reporter cells followed by detection of β-galactosidase activity as previously described [26].

### Immunohistochemistry and In Situ Hybridization

E12.5–E14.5 heads and E18.5 brains were fixed overnight at 4°C in 4% paraformaldehyde and paraffin sections (7 µm) were processed immunohistochemically as described [71]. The primary antibodies included rabbit anti-Raldh3 1∶50 [72], mouse anti-nestin 1∶100 (Millipore), mouse anti-RC2 1∶100 (Developmental Studies Hybridoma Bank at University of Iowa; DSHB), mouse anti-MAP2 1∶200 (Sigma; M4403), rabbit anti-GABA 1∶500 (Millipore), mouse anti-Gad67 1∶50 (Millipore; MAB5406), rabbit anti-GFAP 1∶1000 (Dako), rabbit anti-Foxp1 1∶100 (Abcam), rabbit anti-Ki67 1∶200 (Abcam), rabbit anti-Dlx2 1∶100 (Abcam), mouse anti-VGAT 1∶200 (Synaptic Systems), rabbit anti-VGLUT (Synaptic Systems), and mouse anti-TH (Sigma). In situ hybridization of E18.5 brain sections was performed as described [73] using RARα and RARβ riboprobes.

### Human Embryonic Stem Cell Culture and Differentiation

Human ES cells (line H9) were cultured and passaged weekly on a feeder of irradiated embryonic mouse fibroblasts as described previously [74]. The protocol for RA-induced GABAergic differentiation was based on previous methods [75]. Briefly, human ES cell-derived embryoid bodies (EBs) were cultured in EB growth medium in non-adherent plates for 3 d, followed by RA treatment for 3 d (RAd3). RA was removed and the RAd3 EBs were plated on culture dishes pre-coated with poly-L-ornithine and fibronectin and cultured for an addition 4 d (RAd7) in serum-free neuronal induction medium, comprised of neurobasal medium supplemented with B27, bFGF, and EGF. At RAd7 neuroepithelial rosettes were isolated mechanically from the differentiation cultures with a 2 ml serological pipette. After isolation, rosettes were replated in the same neuronal induction medium for an additional 14 d (RAd21) before fixation.

### Immunocytochemistry

Immunocytochemistry on neurospheres and ES cells was carried out as described [75]. Primary antibodies used included rabbit anti-GABA 1∶1,000 (Millipore), mouse anti-Gad67 1∶200 (Millipore; MAB5406), rabbit anti-GFAP 1∶1,000 (Dako), mouse anti-Tuj1 1∶1,000 (Covance), and guinea pig anti-DCX 1∶500 (Millipore), rabbit anti-Dlx2 1∶200 (Abcam, AB18188), rabbit anti-Foxp1 1∶100 (Abcam), mouse anti-Islet-1 1∶100 (40.2D6) (DSHB), and mouse anti-Lim1/2(4F2) 1∶200 (DSHB).

### Quantitation of Immunoreactivity

Immunopositive cells and total DAPI-stained nuclei were counted to calculate the percentage of immunopositive cells. Five randomly picked areas from three independent experiments were counted for each marker. Data were presented as mean ± SEM; for pair-wise analysis of treatment conditions and/or genotypes, an ANOVA test was used.

## Supporting Information

Figure S1 Dose response of F9 *RARE-lacZ* RA-reporter cell line to RA. (A–E) Cells were cultured in 24-well plates and treated for 18–20 h in serum-free medium with different concentrations of RA ranging from 1 nM to 1000 nM as indicated. Cells were then fixed and assayed for β-galactosidase activity.
(1.96 MB TIF)

Figure S2 Comparison of radial glia proliferation and neurogenesis in absence of RA. (A–B). Double-labeled immunofluorescence was performed on E14.5 forebrain coronal sections of wild-type (WT) and *Raldh3*−/− (R3KO) embryos for radial glia marker nestin and proliferation marker Ki67. (C) Quantification of the ratio of double positive cells in the proliferative zones of the LGE showed that the majority of radial glia are proliferating and demonstrated that *Raldh3*−/− embryos exhibit no defect in radial glia proliferation. (D) Quantification of the number of MAP2-expressing neurons in an equal area of striatum, cortex, and septum from both wild-type and *Raldh3*−/− embryos demonstrates that neurogenesis occurs normally in the mutant forebrains. The percentage was calculated by dividing the total number of DAPI-stained nuclei by the MAP2 immunopositive cell number. Values are listed as mean ± SEM.
(8.88 MB TIF)

Figure S3 Dlx2 and GABA immunoreactivity in embryonic forebrain. (A–B) At E12.5, GABA is expressed in the mantle zones of the MGE and LGE and shows no change in expression in *Raldh3*−/− (R3KO) embryos compared to wild-type (WT). (C–D) At E12.5, Dlx2-expressing cells extend throughout the proliferative zones of both the MGE and LGE (with weaker streams of cells emanating into the cortex) and no change is observed in *Raldh3*−/− embryos. (E–F) At E14.5, *Raldh3*−*/*− embryos continue to exhibit no difference in Dlx2 expression, which has now further expanded. (G–H) At E18.5, the distribution of Dlx2-expressing cells in the cortex appears normal in *Raldh3*−*/*− embryos compared to wild-type. cp, cortical plate; iz, intermediate zone; LGE, lateral ganglionic eminence; m, marginal layer; MGE, medial ganglionic eminence; svz, subventricular zone.
(4.94 MB TIF)

Figure S4 Raldh3-positive cells identified as MAP2-positive neurons in the SVZ. Double-labeled immunofluorescence was performed on wild-type E14.5 forebrain coronal sections at the same rostrocaudal plane as those presented in Figure 4. (A–C) Raldh3 does not colocalize with the radial glia cell marker RC2. (D–F) The majority of Raldh3-positive cells colocalize with the neuronal cell marker MAP2. SVZ, subventricular zone; VZ, ventricular zone.
(4.38 MB TIF)

Figure S5 RA signaling is not required for expression of the vesicular GABA transporter or for differentiation of dopaminergic and glutamatergic neurons in the forebrain. Immunofluorescence was performed on E18.5 forebrain coronal sections of wild-type (WT) and *Raldh3*−/− (KO) embryos. (A–B) Expression of the vesicular GABA transporter (vGAT) is not reduced in mutant forebrain. (C–D) Mutant embryos exhibit normal expression of tyrosine hydroxylase (TH), a marker of dopaminergic neurons, and of vesicular glutamate transporter (VGLUT), a marker of glutamatergic neurons (E–F).
(8.94 MB TIF)

Figure S6 Phenotypic analysis of RA-induced GABAergic neurons derived from human embryonic stem cells. (A–B) In RA-treated cultures, Tuj1-positive neuron populations were immunolabeled with Dlx2, the ventral forebrain marker of GABAergic precursors, whereas no neurons were co-labeled with Foxp1, a marker of GABAergic striatal projection neurons. (C–D) GABA-positive cells were positive for spinal cord interneuron marker Lim1/2, but not for Islet1, a marker of both striatal projection neurons and interneurons of the diencephalon. (E) Quantification of GABA-positive cells that also express either Lim1/2 or Dlx2; as both anti-GABA and anti-Dlx2 were derived in the same species, we employed deconvolution microscopy to distinguish between the nuclear localization of Dlx2 and cytoplasmic localization of GABA; 43.7±11.9 of GABA-positive cells co-labeled with Lim1/2, and 37.5±11.5 were co-labeled with Dlx2.
(4.82 MB TIF)

Figure S7 Loss of RA synthesis in the meninges does not affect radial expansion of the cortex. (A–B) MAP2 labeling of postmitotic neuronal layer. Arrows (same length) demonstrate that *Raldh2*−/− mutant does not exhibit a change in medial-lateral width of the dorsal ventricular zone (vz). (C–D) MAP2 labeling shows that *Raldh2*−/− mutant has normal radial expansion of cortex (cx). (E–F) RC2 (radial glia) labeling; mutant has normal radial expansion of dorsal cortex.
(2.87 MB TIF)

Figure S8 Expression pattern of RARα and RARβ in embryonic forebrain. Representative coronal sections are illustrated for each gene examined by in situ hybridization in wild-type embryos at E18.5. (A) RARα is expressed both in the developing striatum and cortex. (B) RARβ transcripts were highly expressed striatum but not the cortex. Neither gene was expressed in the ventricular zone. Cx, cortex; vz, ventricular zone.
(3.42 MB TIF)

## Abbreviations

ES cell
embryonic stem cell
GABA: gamma-aminobutyric acid
Gad67: glutamic acid decarboxylase
GFAP: glial fibrillary acidic protein
LGE: lateral ganglionic eminence
MAP2: Microtubule-associated protein 2
MGE: medial ganglionic eminence
RA: retinoic acid
Raldh2: retinaldehyde dehydrogenase 2
Raldh3: retinaldehyde dehydrogenase 3
RAR: retinoic acid receptor
Rdh10: retinol dehydrogenase 10
SVZ: subventricular zone
Tuj1: β-tubulin-III
VGAT: vesicular GABA transporter
VGLUT: vesicular glutamate transporter
