# Supplementary figures and images for: Retinoic Acid Functions as a Key GABAergic Differentiation Signal in the Basal Ganglia
Source: PLoS Biol. 2011 Apr 12;9(4):e1000609. doi: 10.1371/journal.pbio.1000609 (PMC3075211; doi:10.1371/journal.pbio.1000609)

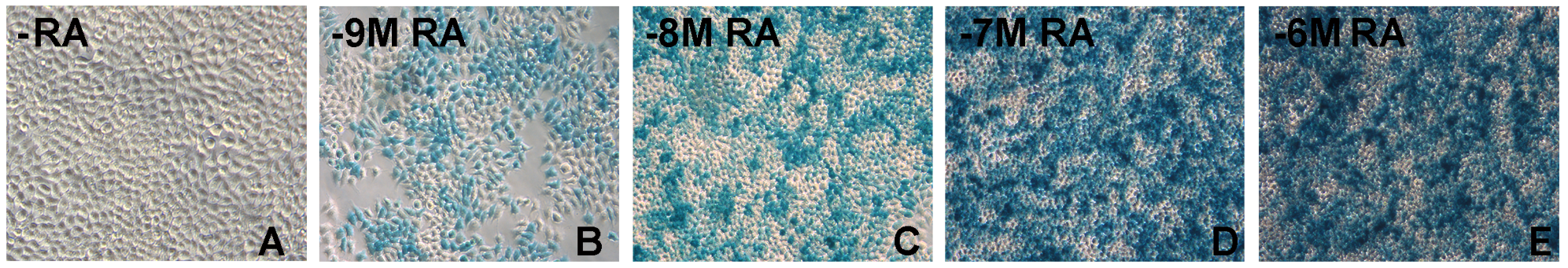

Supplement: Figure S1 — Dose response of F9 RARE-lacZ RA-reporter cell line to RA. (A–E) Cells were cultured in 24-well plates and treated for 18–20 h in serum-free medium with different concentrations of RA ranging from 1 nM to 1000 nM as indicated. Cells were then fixed and assayed for β-galactosidase activity. (1.96 MB TIF) [file pbio.1000609.s001.tif]

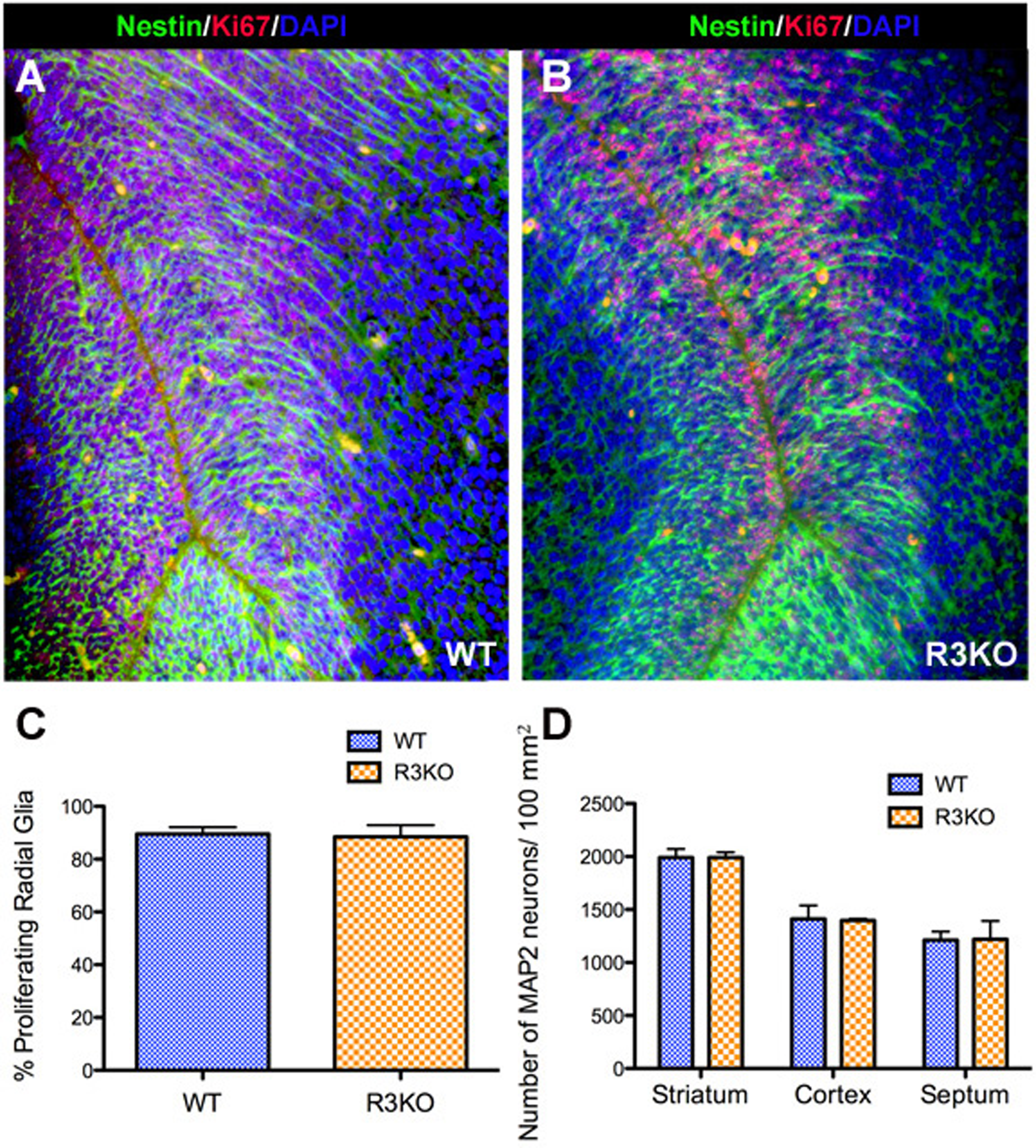

Supplement: Figure S2 — Comparison of radial glia proliferation and neurogenesis in absence of RA. (A–B). Double-labeled immunofluorescence was performed on E14.5 forebrain coronal sections of wild-type (WT) and Raldh3−/− (R3KO) embryos for radial glia marker nestin and proliferation marker Ki67. (C) Quantification of the ratio of double positive cells in the proliferative zones of the LGE showed that the majority of radial glia are proliferating and demonstrated that Raldh3−/− embryos exhibit no defect in radial glia proliferation. (D) Quantification of the number of MAP2-expressing neurons in an equal area of striatum, cortex, and septum from both wild-type and Raldh3−/− embryos demonstrates that neurogenesis occurs normally in the mutant forebrains. The percentage was calculated by dividing the total number of DAPI-stained nuclei by the MAP2 immunopositive cell number. Values are listed as mean ± SEM. (8.88 MB TIF) [file pbio.1000609.s002.tif]

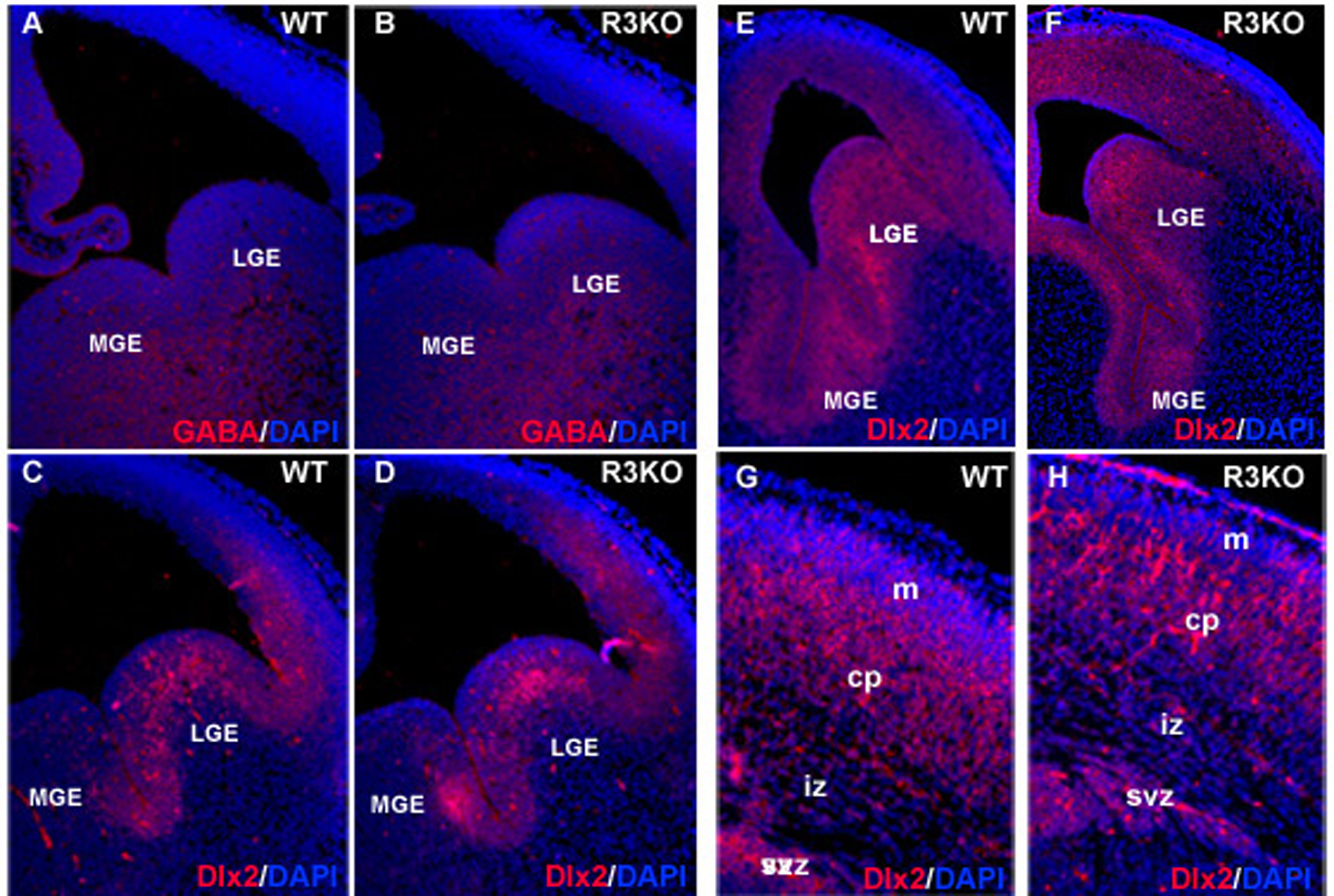

Supplement: Figure S3 — Dlx2 and GABA immunoreactivity in embryonic forebrain. (A–B) At E12.5, GABA is expressed in the mantle zones of the MGE and LGE and shows no change in expression in Raldh3−/− (R3KO) embryos compared to wild-type (WT). (C–D) At E12.5, Dlx2-expressing cells extend throughout the proliferative zones of both the MGE and LGE (with weaker streams of cells emanating into the cortex) and no change is observed in Raldh3−/− embryos. (E–F) At E14.5, Raldh3−/− embryos continue to exhibit no difference in Dlx2 expression, which has now further expanded. (G–H) At E18.5, the distribution of Dlx2-expressing cells in the cortex appears normal in Raldh3−/− embryos compared to wild-type. cp, cortical plate; iz, intermediate zone; LGE, lateral ganglionic eminence; m, marginal layer; MGE, medial ganglionic eminence; svz, subventricular zone. (4.94 MB TIF) [file pbio.1000609.s003.tif]

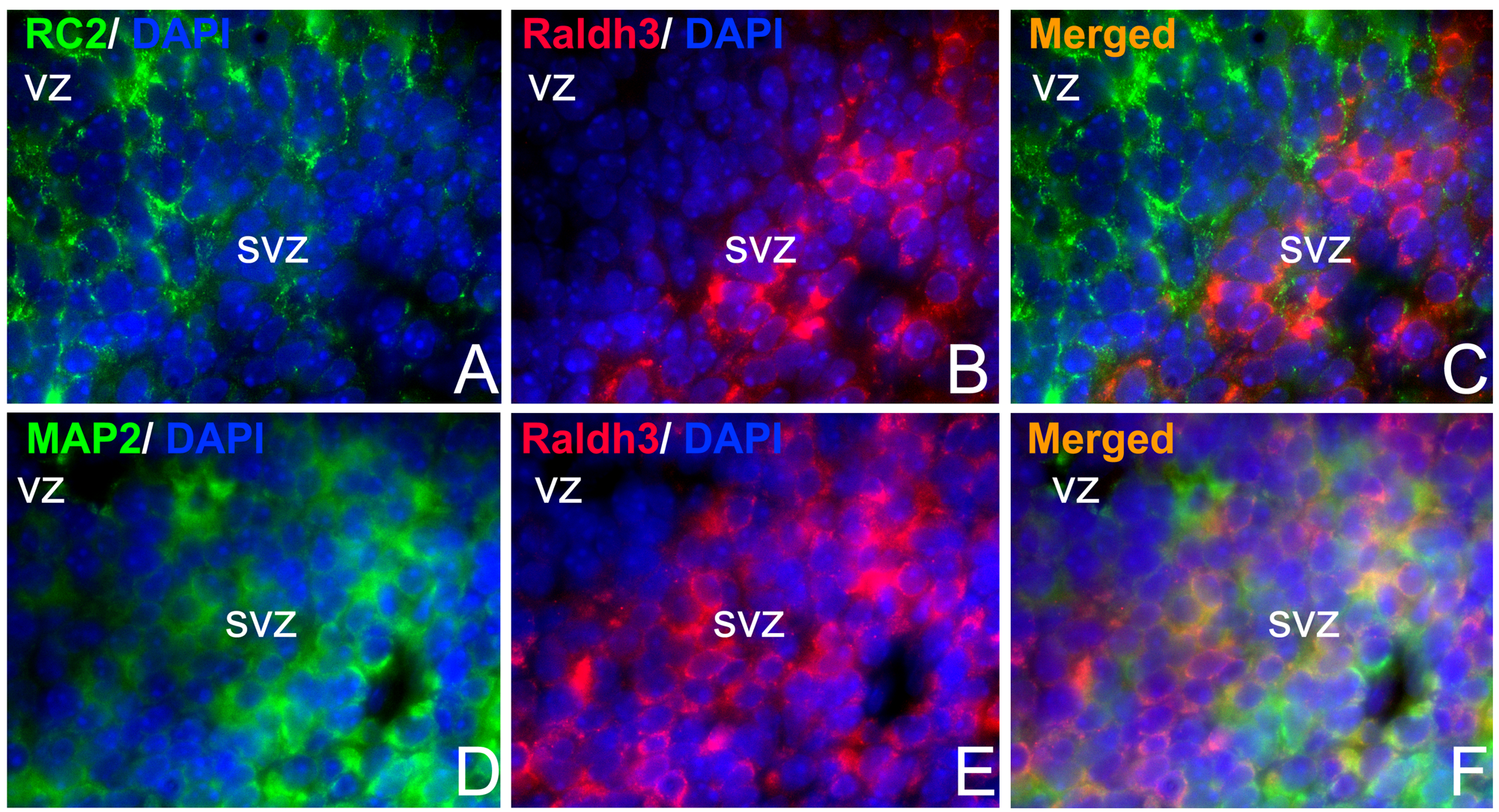

Supplement: Figure S4 — Raldh3-positive cells identified as MAP2-positive neurons in the SVZ. Double-labeled immunofluorescence was performed on wild-type E14.5 forebrain coronal sections at the same rostrocaudal plane as those presented in Figure 4. (A–C) Raldh3 does not colocalize with the radial glia cell marker RC2. (D–F) The majority of Raldh3-positive cells colocalize with the neuronal cell marker MAP2. SVZ, subventricular zone; VZ, ventricular zone. (4.38 MB TIF) [file pbio.1000609.s004.tif]

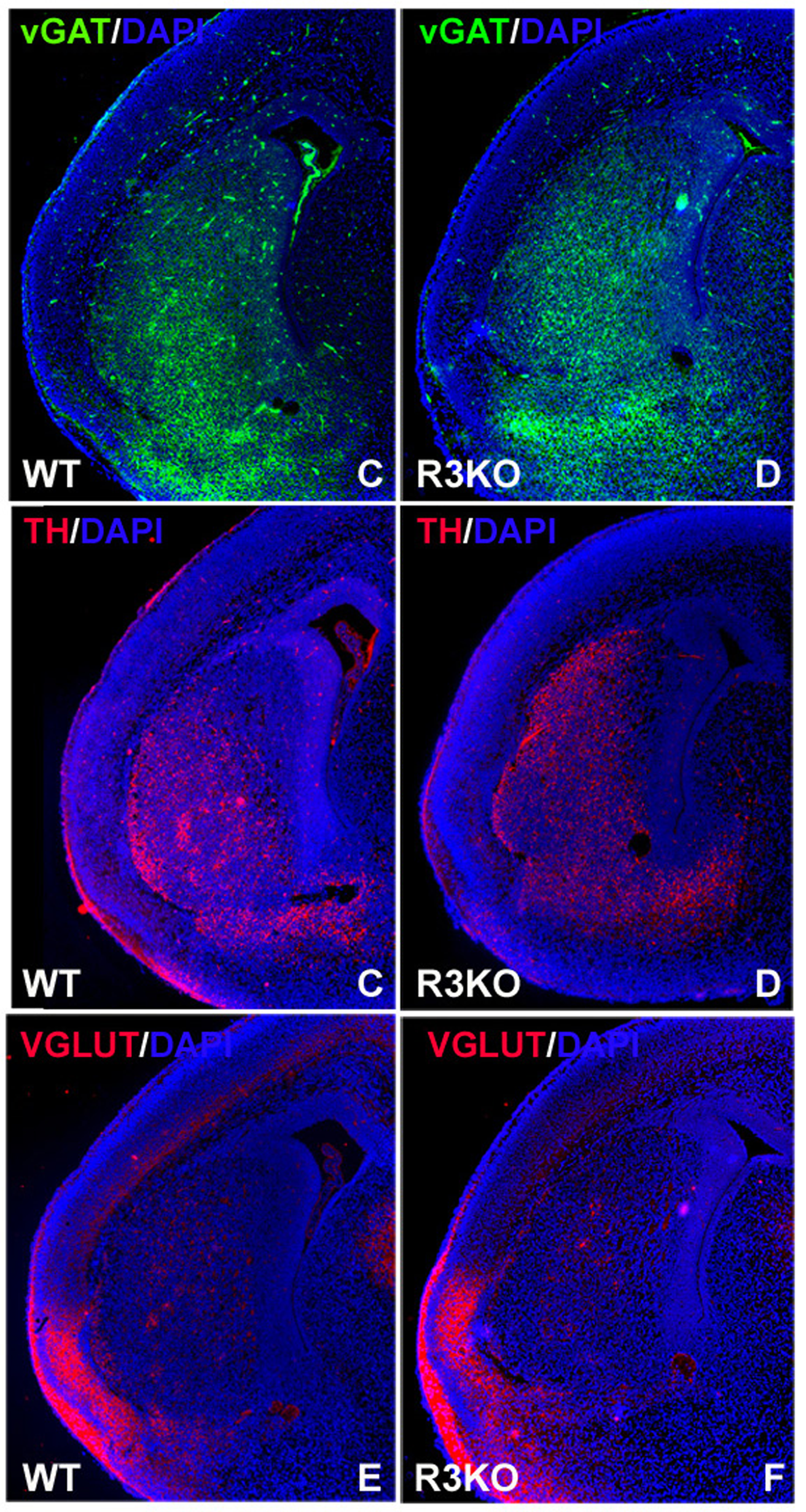

Supplement: Figure S5 — RA signaling is not required for expression of the vesicular GABA transporter or for differentiation of dopaminergic and glutamatergic neurons in the forebrain. Immunofluorescence was performed on E18.5 forebrain coronal sections of wild-type (WT) and Raldh3−/− (KO) embryos. (A–B) Expression of the vesicular GABA transporter (vGAT) is not reduced in mutant forebrain. (C–D) Mutant embryos exhibit normal expression of tyrosine hydroxylase (TH), a marker of dopaminergic neurons, and of vesicular glutamate transporter (VGLUT), a marker of glutamatergic neurons (E–F). (8.94 MB TIF) [file pbio.1000609.s005.tif]

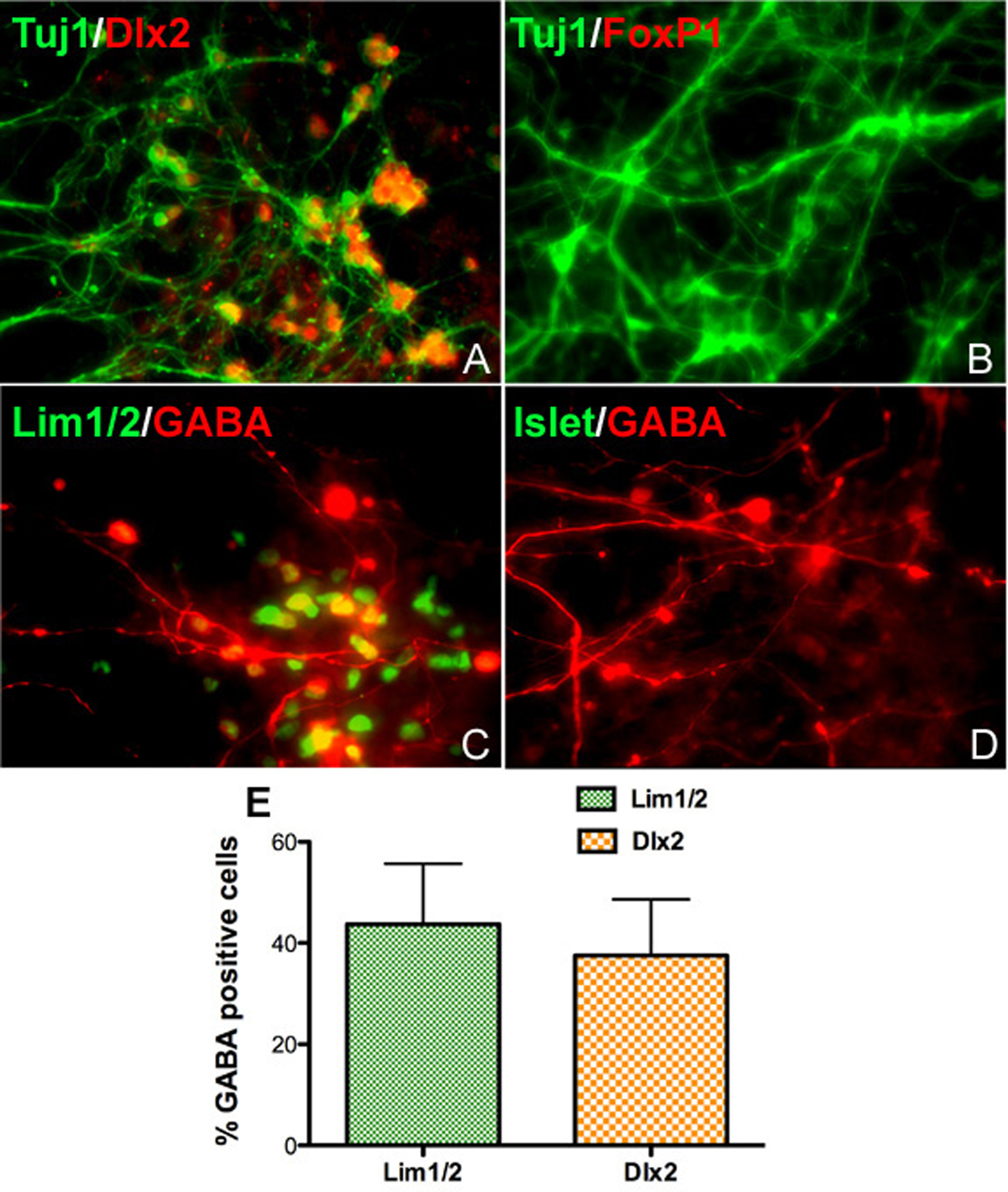

Supplement: Figure S6 — Phenotypic analysis of RA-induced GABAergic neurons derived from human embryonic stem cells. (A–B) In RA-treated cultures, Tuj1-positive neuron populations were immunolabeled with Dlx2, the ventral forebrain marker of GABAergic precursors, whereas no neurons were co-labeled with Foxp1, a marker of GABAergic striatal projection neurons. (C–D) GABA-positive cells were positive for spinal cord interneuron marker Lim1/2, but not for Islet1, a marker of both striatal projection neurons and interneurons of the diencephalon. (E) Quantification of GABA-positive cells that also express either Lim1/2 or Dlx2; as both anti-GABA and anti-Dlx2 were derived in the same species, we employed deconvolution microscopy to distinguish between the nuclear localization of Dlx2 and cytoplasmic localization of GABA; 43.7±11.9 of GABA-positive cells co-labeled with Lim1/2, and 37.5±11.5 were co-labeled with Dlx2. (4.82 MB TIF) [file pbio.1000609.s006.tif]

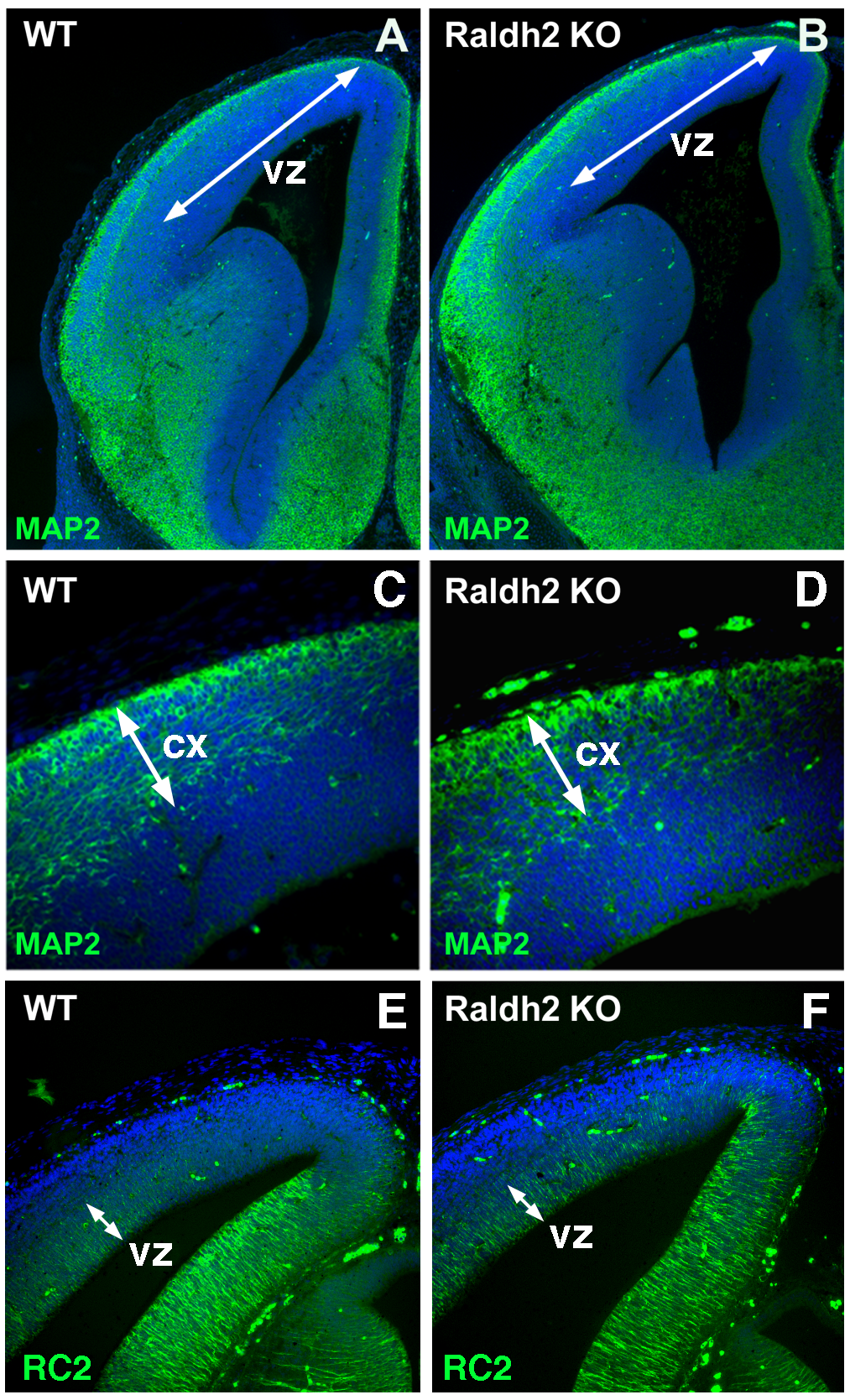

Supplement: Figure S7 — Loss of RA synthesis in the meninges does not affect radial expansion of the cortex. (A–B) MAP2 labeling of postmitotic neuronal layer. Arrows (same length) demonstrate that Raldh2−/− mutant does not exhibit a change in medial-lateral width of the dorsal ventricular zone (vz). (C–D) MAP2 labeling shows that Raldh2−/− mutant has normal radial expansion of cortex (cx). (E–F) RC2 (radial glia) labeling; mutant has normal radial expansion of dorsal cortex. (2.87 MB TIF) [file pbio.1000609.s007.tif]

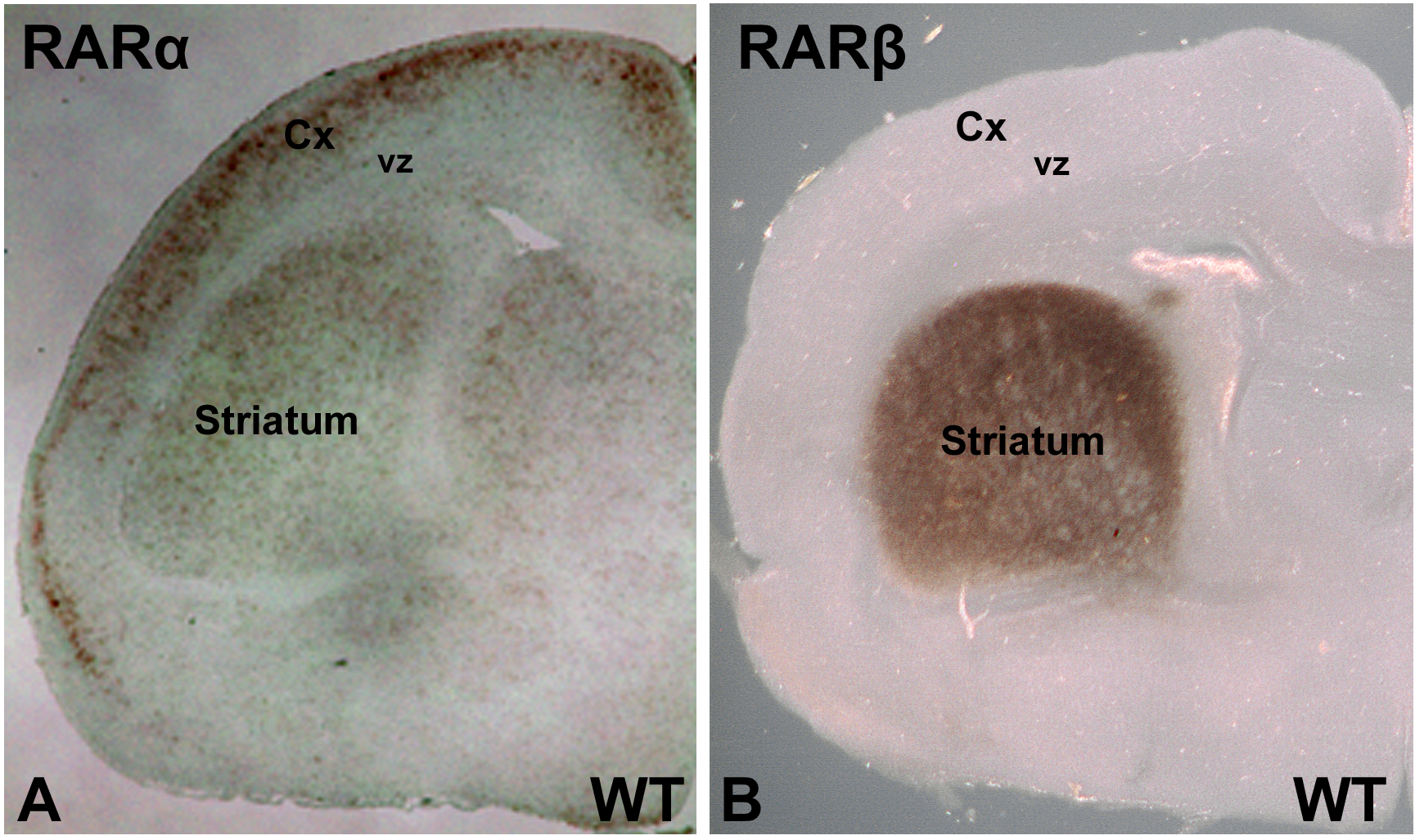

Supplement: Figure S8 — Expression pattern of RARα and RARβ in embryonic forebrain. Representative coronal sections are illustrated for each gene examined by in situ hybridization in wild-type embryos at E18.5. (A) RARα is expressed both in the developing striatum and cortex. (B) RARβ transcripts were highly expressed striatum but not the cortex. Neither gene was expressed in the ventricular zone. Cx, cortex; vz, ventricular zone. (3.42 MB TIF) [file pbio.1000609.s008.tif]
